# Biomimetic Supramolecular Assembly with IGF‐1C Delivery Ameliorates Inflammatory Bowel Disease (IBD) by Restoring Intestinal Barrier Integrity

**DOI:** 10.1002/advs.202403075

**Published:** 2024-07-23

**Authors:** Enze Fu, Meng Qian, Ningning He, Yilun Yin, Yue Liu, Zhibo Han, ZhongChao Han, Qiang Zhao, Xiaocang Cao, Zongjin Li

**Affiliations:** ^1^ School of Medicine Nankai University Tianjin 300071 China; ^2^ Key Laboratory of Bioactive Materials Ministry of Education College of Life Sciences Nankai University Tianjin 300071 China; ^3^ Tianjin Key Laboratory of Human Development and Reproductive Regulation Tianjin Central Hospital of Gynecology Obstetrics Nankai University Affiliated Hospital of Obstetrics and Gynecology Tianjin 300052 China; ^4^ Tianjin Key Laboratory of Radiation Medicine and Molecular Nuclear Medicine Institute of Radiation Medicine Chinese Academy of Medical Science and Peking Union Medical College Tianjin 300192 China; ^5^ Academy of Medical Engineering and Translational Medicine Tianjin University Tianjin 300072 China; ^6^ Jiangxi Engineering Research Center for Stem Cells Shangrao 334109 China; ^7^ Tianjin Key Laboratory of Engineering Technologies for Cell Pharmaceuticals National Engineering Research Center of Cell Products AmCellGene Co., Ltd. Tianjin 300457 China; ^8^ Department of Hepato‐Gastroenterology Tianjin Medical University General Hospital Tianjin Medical University Tianjin 300050 China; ^9^ Henan Key Laboratory of Cardiac Remodeling and Transplantation Zhengzhou Seventh People's Hospital Zhengzhou 450016 China; ^10^ National Key Laboratory of Kidney Diseases Chinese PLA General Hospital Beijing 100853 China

**Keywords:** C domain peptide of insulin‐like growth factor‐1 (IGF‐1C), inflammatory bowel disease (IBD), intestinal barrier integrity, microbiome homeostasis, supramolecular assembly

## Abstract

The management of dysfunctional intestinal epithelium by promoting mucosal healing and modulating the gut microbiota represents a novel therapeutic strategy for inflammatory bowel disease (IBD). As a convenient and well‐tolerated method of drug delivery, intrarectal administration may represent a viable alternative to oral administration for the treatment of IBD. Here, a biomimetic supramolecular assembly of hyaluronic acid (HA) and β‐cyclodextrin (HA‐β‐CD) for the delivery of the C domain peptide of insulin‐like growth factor‐1 (IGF‐1C), which gradually releases IGF‐1C, is developed. It is identified that the supramolecular assembly of HA‐β‐CD enhances the stability and prolongs the release of IGF‐1C. Furthermore, this biomimetic supramolecular assembly potently inhibits the inflammatory response, thereby restoring intestinal barrier integrity. Following HA‐β‐CD‐IGF‐1C administration, 16S rDNA sequencing reveals a significant increase in the abundance of the probiotic *Akkermansia*, suggesting enhanced intestinal microbiome homeostasis. In conclusion, the findings demonstrate the promise of the HA‐based mimicking peptide delivery platform as a therapeutic approach for IBD. This biomimetic supramolecular assembly effectively ameliorates intestinal barrier function and intestinal microbiome homeostasis, suggesting its potential for treating IBD.

## Graphical abstract

The management of dysfunctional intestinal epithelium by promoting mucosal healing and modulating gut microbiota represents a promise therapeutic strategy for inflammatory bowel disease (IBD). Enema administration of hyaluronic acid (HA)‐based supramolecular assembly delivering IGF‐1C alleviates IBD by reducing inflammatory response, thereby restoring epithelial integrity. The findings highlight the potential of this HA‐based platform for IBD treatment.

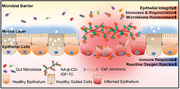

## 1 Introduction

Inflammatory bowel disease (IBD), including Crohn's disease (CD) and ulcerative colitis (UC), is characterized by the pathogenesis of persistent diarrhea, abdominal pain, bleeding ulcers, and bloody stool caused by a variety of factors related to environmental, microbial, genetic, and immunological factors and affects the whole gastrointestinal tract.^[^ ^1^ ^]^ In the early stages of IBD, a dysregulated epithelial barrier allows for increased infiltration of pathogenic microorganisms and triggers the production of reactive oxygen species (ROS) by recruited inflammatory cells.^[^ ^2^ ^]^ This cycle of inflammation and barrier dysfunction further leads to epithelial deficiency, increasing susceptibility to invasion and impairing intestinal function, including the delicate balance of the gut microbiome.^[^ ^2^, ^3^ ^]^

Increasing evidence suggests that both endogenous and exogenous insulin‐like growth factor 1 (IGF‐1) serve as pro‐mitogenic and macrophage‐regulated proteins, contributing to the immunomodulatory characteristics of mesenchymal stem cells (MSCs) and promoting the integrity of crypt cells.^[^ ^4^ ^]^ Functional synthetic polypeptide drugs with a molecular weight less than 10 kDa and composed of 10–100 amino acids combined with appropriate delivery systems, such as hydrogels and bioactive patches, have been developed to improve selectivity and biocompatibility while reducing synthetic costs and have shown efficacy similar to that of native factors.^[^ ^5^ ^]^ For example, the active C domain of IGF‐1 (IGF‐1C) containing GYGSSSRRAPQT peptides and its derived hydrogel has been applied in the treatment of acute kidney injury.^[^ ^6^ ^]^

Supramolecular chemistry has emerged as a promising field for developing novel materials and therapeutics in various areas, including tissue engineering and injectable drug delivery. One of the most extensively studied examples of supramolecular chemistry is the host–guest interaction between β‐cyclodextrin and adamantane (β‐CD‐Ad).^[^ ^7^ ^]^ The resulting β‐CD‐Ad inclusion complexes possess unique properties, such as improved solubility, stability, and bioavailability.^[^ ^8^ ^]^ Noncovalent interactions between β‐cyclodextrin and adamantane provide a versatile means of achieving precise control over the release rate and localization of guest molecules.^[^ ^9^ ^]^ As such, the incorporation of β‐CD‐Ad inclusion complexes into biomaterials offers a powerful strategy to achieve targeted drug delivery and tissue regeneration and has great potential for a wide range of biomedical applications.^[^ ^10^ ^]^

Intrarectal administration offers several advantages for the treatment of IBD in a clinical setting. One of the most significant benefits is the ability to bypass first‐pass metabolism in the liver, leading to rapid drug absorption by the intestinal mucosa. In addition, intrarectal administration can effectively prevent drug exposure to the acidic environment of the stomach, reducing the risk of degradation while simultaneously improving bioavailability. Here, we developed an IGF‐1C modified by adamantane at the N‐terminus (Ad‐IGF‐1C) and anchored it to β‐cyclodextrin‐grafted hyaluronic acid (HA‐β‐CD) to generate the supramolecular assembly of HA‐β‐CD‐IGF‐1C. We hypothesized that enema administration of the supramolecular assembly would have a protective effect in mice with IBD. Our results showed that the supramolecular assembly of HA‐β‐CD‐IGF‐1C significantly decreased inflammation and ameliorated intestinal barrier function. Furthermore, we performed 16S rDNA sequencing and further confirmed that HA‐β‐CD‐IGF‐1C altered the composition and function of gut microbiome. Overall, enema administration of HA‐based supramolecular assembly delivering IGF‐1C effectively alleviated IBD by reducing inflammatory response, thereby restoring epithelial integrity. These findings highlight the potential of this HA platform for treating IBD.

## 2 Results

### 2.1 Design and Characteristics of the Supramolecular Assembly of HA‐β‐CD‐IGF‐1C

**Figure 1:**
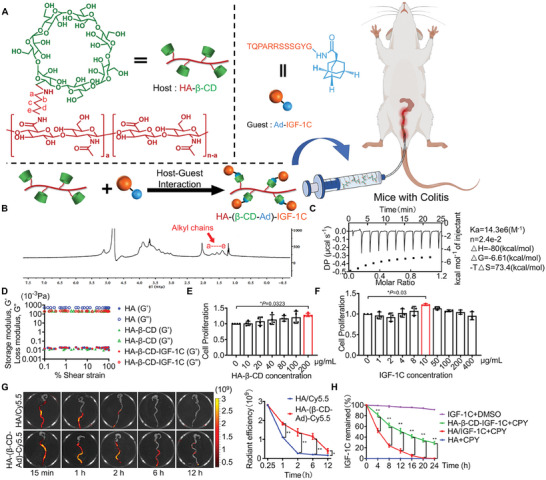
Design and characterization of the supramolecular assembly of HA‐β‐CD‐IGF‐1C. A) Schematic overview of the preparation of the supramolecular assembly of HA‐β‐CD‐IGF‐1C enabling delivery of IGF‐1C by intrarectal administration. B) ^1^H NMR spectroscopy of HA‐β‐CD was used in this study. C) ITC titration experiment of HA‐β‐CD with Ad‐IGF‐1C in PBS solution. D) The rheological profiles of HA, HA‐β‐CD, and the HA‐β‐CD‐IGF‐1C system with changes in frequency sweep were determined by analyzing the storage modulus (*G*′) and the loss modulus (*G*″). E,F) CCK‐8 assay showing the proliferation of MODE‐K cells pretreated with a wide range of concentrations of HA‐β‐CD and Ad‐IGF‐1C for 48 h. G) NIR imaging and quantitative analysis of colorectal tissues after intrarectal administration of HA/Cy5.5 or HA‐(β‐CD‐Ad)‐Cy5.5. H) The percentage of IGF‐1C remaining after treatment with 1 mg mL^−1^ carboxypeptidase Y (CPY) at different incubation times. Free IGF‐1C with DMSO or HA with CPY was used as a control. All data are expressed as mean ± s.d. The *P*‐values were calculated using one‐way ANOVA with Tukey's HSD multiple comparison post hoc test and defined as *P < 0.05 and **P < 0.01. Scale bar, 300 µm.

Host–guest chemistry has been regarded as a prospective delivery system in supramolecular theranostics. To combine this advantage, we developed HA‐β‐CD and Ad‐IGF‐1C to generate guest–host pairs (HA‐β‐CD‐IGF‐1C) that can slowly release IGF‐1C at the target position (**Figure** **1**A). The presence of alkyl chains in HA and HA‐β‐CD was confirmed by ^1^H NMR spectroscopy (Figure 1B). Then, the interaction between HA‐β‐CD and Ad‐IGF‐1C in PBS was investigated by isothermal titration calorimetry (ITC). The ITC also confirmed that the association constant Ka was 14.3 × 10^6^ m ^−1^, which indicated a notable binding affinity (Figure 1C). Furthermore, it is critical to understand the viscoelastic behavior of the supramolecular assembly of HA‐β‐CD‐IGF‐1C. We performed rheological tests, and the results revealed that the mechanical properties of HA‐β‐CD‐IGF‐1C were not significantly altered by the addition of Ad‐IGF‐1C to HA‐β‐CD. Our data revealed that the loss modulus (*G*″) was greater than the storage modulus (*G*′) at 37 °C, indicating that both HA and HA‐β‐CD‐IGF‐1C exhibited viscous behavior and are suitable for enema administration (Figure 1D). Moreover, a Cell Counting Kit‐8 (CCK‐8) assay of intestinal epithelial MODE‐K cells indicated that HA‐β‐CD alone had an optimal effect on cell proliferation at 200 µg mL^−1^, and the optimal concentration of Ad‐IGF‐1C was 10 µg mL^−1^ (Figure 1E,F). We hypothesized that the biocompatible and fluid nature of our HA‐β‐CD‐IGF‐1C would confer adaptability to the peristaltic movement and enzymatic conditions prevailing in the colonic environment and ensure sufficient intestinal retention for sustained IGF‐1C release. The contribution of β‐CD and Ad supramolecular interactions to the bioadhesive properties of coatings on the gastrointestinal mucosa was investigated in vivo using the Cy5.5‐modified near infrared fluorescent dye adamantane. Mice were administered a single intrarectal enema of 0.2 mL of HA‐(β‐CD‐Ad)‐Cy5.5 and were euthanized at 0.25, 1, 2, 6, or 12 h to assess fluorescence retention. HA/Cy5.5 exhibited limited retention within the colorectal tract for a maximum of 2 hours. In contrast, HA‐(β‐CD‐Ad)‐Cy5.5 demonstrated retention for at least 12 h (Figure 1G). Next, we evaluated the protective effect of HA‐β‐CD and the biodegradability of HA‐β‐CD‐IGF‐1C under conditions that mimic the intestinal environment. Consistent with its in vivo bioadhesive property, HA‐β‐CD‐IGF‐1C was found to be more resistant to carboxypeptidase Y (CPY)‐mediated degradation than free IGF‐1C (Figure 1H). All these results indicate that HA‐β‐CD‐IGF‐1C exhibited suitable mechanical properties, biocompatibility, and adhesion to the gastrointestinal mucosa, ensuring sustained drug release and enhanced retention within the colorectal tract.

### 2.2 HA‐β‐CD‐IGF‐1C Ameliorates IBD

**Figure 2:**
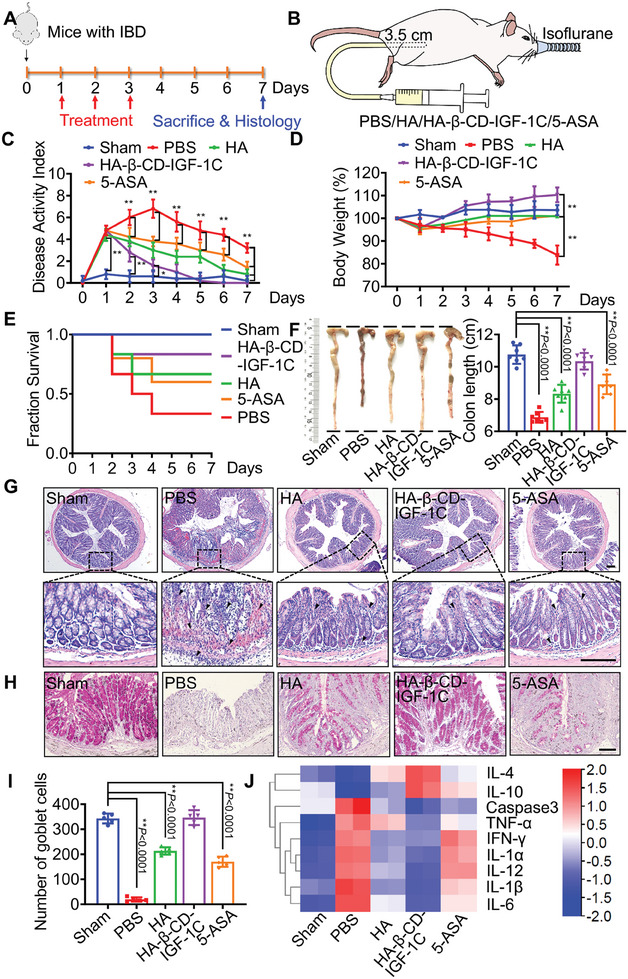
Therapeutic effect of the supramolecular assembly of HA‐β‐CD‐IGF‐1C. A) Experimental design to determine the therapeutic effect of the supramolecular assembly of HA‐β‐CD‐IGF‐1C. B) Experimental schema of enema administration. C) DAI scores of healthy or colitis mice. D) The percentage of changes in body weight. E) Kaplan–Meier survival analysis of mice. F) Representative images of the appearance of the colon tissues and colon length. G) H&E images of colon tissues. Arrows: inflammatory sites. H,I) PAS staining images illustrate the number of goblet cells in the colonic tissues. J) Heatmap of genes related to inflammation determined by RT‐qPCR. All data are expressed as mean ± s.d. The *P*‐values were calculated using one‐way ANOVA with Tukey's HSD multiple comparison post hoc test and defined as *P < 0.05 and **P < 0.01. All experiments were performed more than three times. Scale bar, 100 µm.

To evaluate the therapeutic efficacy of HA‐β‐CD‐IGF‐1C against IBD, we first used a mouse model of Crohn's disease induced by intrarectal administration of TNBS (2,4,6‐trinitro‐benzene sulfonic acid). As shown in **Figure** **2**A, BALB/c mice were continuously treated with HA, HA‐β‐CD‐IGF‐1C or a clinically approved drug mesalazine (5‐ASA) for 3 d, and body weight and DAI (disease activity index) were monitored daily according to Table S1 (Supporting Information). A soft catheter designed for small animals was used for intrarectal administration of HA‐β‐CD‐IGF‐1C (Figure 2B). The results of DAI and body weight revealed that HA‐β‐CD‐IGF‐1C markedly reduced the severity of symptoms of colitis (Figure 2C,D). Kaplan–Meier survival analysis demonstrated that HA‐CD‐IGF‐1C treatment led to a significant improvement in the survival rate of mice with colitis (Figure 2E). Furthermore, we investigated the whole colorectal length and histomorphological profile on day 7. HA‐β‐CD‐IGF‐1C‐treated mice showed reduced inflammatory cell infiltration in physiological structures (Figure 2F,G). Periodic acid‐Schiff (PAS) staining revealed that mucoprotein‐containing goblet cells in the colonic epithelium recovered significantly (Figure 2H,I). The therapeutic effect of HA‐β‐CD‐IGF‐1C on UC model induced by dextran sulfate sodium (DSS) was consistent to Crohn's disease model induced by TNBS (Figure S1A–E, Supporting Information). The expression levels of inflammatory factors and apoptotic gene were evaluated using RT‐qPCR and ELISA. Interestingly, both HA and HA‐β‐CD‐IGF‐1C treatment resulted in lower levels of apoptosis and pro‐inflammatory cytokines. Notably, HA‐β‐CD‐IGF‐1C exhibited the best performance (Figure 2J and Figure S2, Supporting Information). Collectively, these findings suggest that HA‐β‐CD‐IGF‐1C exhibited superior therapeutic efficacy compared to the clinically approved drug 5‐ASA, highlighting its potential as a promising treatment for IBD.

### 2.3 HA‐β‐CD‐IGF‐1C Reduces ROS Production

**Figure 3:**
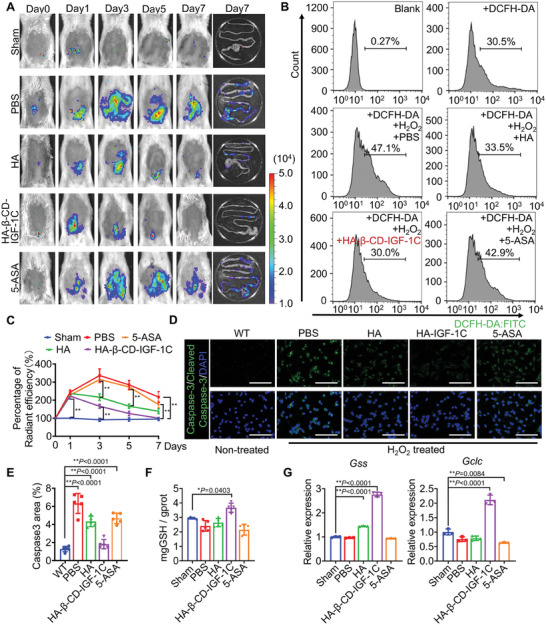
The supramolecular assembly of HA‐β‐CD‐IGF‐1C ameliorates ROS accumulation. A) ROS activity in colitis mice was monitored by an IVIS Lumina imaging system at the indicated time points. B) The oxidized DCF fluorescence of MODE‐K cells was measured by flow cytometry after PBS/HA/HA‐β‐CD‐IGF‐1C/5‐ASA treatment and H_2_O_2_ exposure. C) Quantitative analysis of the intensity of bioluminescence signal at the indicated time points. D) Representative immunofluorescence images of total Caspase‐3. E) Quantitative analysis of signal intensity showing the antiapoptotic effect of HA‐β‐CD‐IGF‐1C. Cell nuclei were stained with DAPI (blue). Representative images are shown from six slides in three independent experiments. F) Total glutathione (GSH) content of the colon tissues. G) RT‐qPCR analysis showing the expression of GSH‐related upstream genes in colon tissues. All data are expressed as the mean ± s.d. The *P*‐values were calculated using one‐way ANOVA with Tukey's HSD multiple comparison post hoc test and defined as *P < 0.05 and **P < 0.01. Scale bar, 100 µm.

Elevated levels of ROS have been shown to impede the healing process of IBD.^[^ ^11^ ^]^ Here, we investigated the ability of HA‐β‐CD‐IGF‐1C to scavenge ROS during colitis using bioluminescence imaging (BLI). HA‐β‐CD‐IGF‐1C significantly decreased the levels of ROS (**Figure** **3**A,C). In addition, we investigated whether HA‐β‐CD‐IGF‐1C also had ROS scavenging performance in vitro. A 2′,7′‐dichlorofluorescein diacetate (DCFH‐DA) staining assay was performed and quantified by flow cytometry as previously described.^[^ ^12^ ^]^ Treatment with HA‐β‐CD‐IGF‐1C after exposure to H_2_O_2_ resulted in a greater reduction in the proportion of DCF fluorescence positive cells (Figure 3B). Furthermore, we generated an intestinal cell line that expressed firefly luciferase (Fluc) and green fluorescence protein (GFP) to further investigate cell viability (Figure S3, Supporting Information). BLI results revealed that HA‐β‐CD‐IGF‐1C ameliorated cell apoptosis (Figure S4, Supporting Information). Immunostaining and Western blot assay also confirmed the anti‐apoptotic effects of HA‐β‐CD‐IGF‐1C (Figure 3D,E and Figure S5, Supporting Information). Moreover, our results demonstrated that the administration of HA‐β‐CD‐IGF‐1C significantly increased glutathione (GSH) (Figure 3F), a pivotal antioxidant known for its anti‐inflammatory properties.^[^ ^13^ ^]^ We also observed the upregulation of the catalyze GSH productions, glutamate‐cysteine ligase catalytic subunit (*Gclc*) and glutathione synthetase (*Gss*) (Figure 3G). However, free IGF‐1 exhibited minimal therapeutic efficacy (Figure S6, Supporting Information), and this may attribute to its extremely short half‐life.

### 2.4 HA‐β‐CD‐IGF‐1C Restores Intestinal Epithelial Integrity

**Figure 4:**
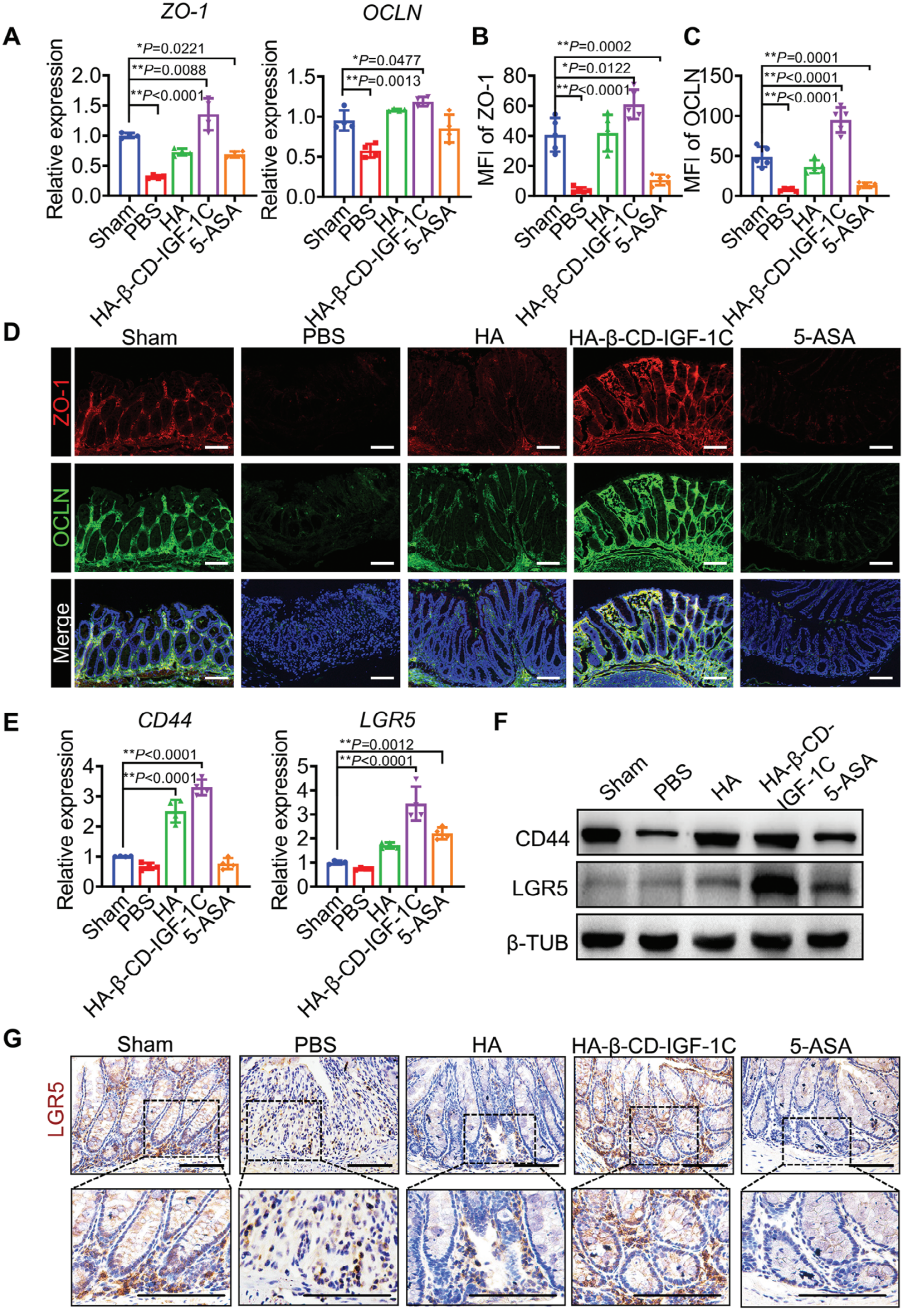
The supramolecular assembly of HA‐β‐CD‐IGF‐1C contributes to the integrity of the intestinal epithelium. A) RNA expression of genes associated with intestinal epithelial integrity by RT‐qPCR. B) Representative immunofluorescence images and C,D) quantitative analysis of OCLN (green) and ZO‐1 (red) showing the degree of intestinal barrier integrity in colon tissues. Cell nuclei were stained with DAPI (blue). E) RNA expression of genes associated with intestinal epithelial stemness by RT‐qPCR. F) Western blot analysis of LGR5 and CD44 in the mouse epithelium under the indicated conditions. β‐Tubulin was included as a loading control. G) Representative image of LGR5 immunohistochemical staining. The positive cells were brown and the distribution of positive cells in the colon was mainly localized at the bottom of the crypts. Representative images are shown from six slides in three independent experiments. All data are expressed as the mean ± s.d. The *P*‐values were calculated using one‐way ANOVA with Tukey's HSD multiple comparison post hoc test and defined as *P < 0.05 and **P < 0.01. Scale bar, 100 µm.

The colonic epithelium serves as a barrier that is essential for host microbial homeostasis.^[^ ^14^ ^]^ To investigate if supramolecular assembly of HA‐β‐CD‐IGF‐1C can ameliorate the inflammation‐induced dysfunctional epithelium, we examined the tight junction‐associated zonula occluden (*ZO‐1*), occludin (*OCLN*) and the epithelial junctional adhesion molecule E‐cadherin (*E‐cad*). The results showed that these molecules were upregulated with HA‐β‐CD‐IGF‐1C treatment (**Figure** **4**A–D and Figures S7 and S8, Supporting Information). We further investigated the mucosal protective effect of HA‐β‐CD‐IGF‐1C on colonic barrier function in vitro by measuring the concentration of 4 kDa fluorescein isothiocyanate (FITC)‐dextran (FD4) (Figure S9A, Supporting Information), which was filtered through a Caco‐2 monolayer seeded on a 0.4 µm transwell insert as previously described.^[^ ^15^ ^]^ This result demonstrated that HA‐β‐CD‐IGF‐1C had the greatest protective effects in vitro (Figure S9B). Consistent with these results, we also observed fewer FD4 signals in the blood of mice treated with HA‐β‐CD‐IGF‐1C (Figure S10A,B). Furthermore, we directly detected the degree of leakage of the colonic barrier using an IVIS Lumina imaging system and found that the intensity of the FD4 signal was significantly reduced in the HA‐β‐CD‐IGF‐1C group (Figure S10C,D). HA is a major ligand of CD44, which is highly expressed in intestinal stem cells. We further confirmed that HA‐β‐CD‐IGF‐1C can increase the expression of CD44 and LGR5, which are markers of intestinal stem cell (Figure 4E–G). Moreover, HA‐β‐CD‐IGF‐1C treatment resulted in a notable increase of cell proliferation at the basal region of the intestinal crypts (Figure S11, Supporting Information).

### 2.5 HA‐β‐CD‐IGF‐1C Alters the Composition of the Gut Microbiota and Increases *Akkermansia* Abundance

**Figure 5:**
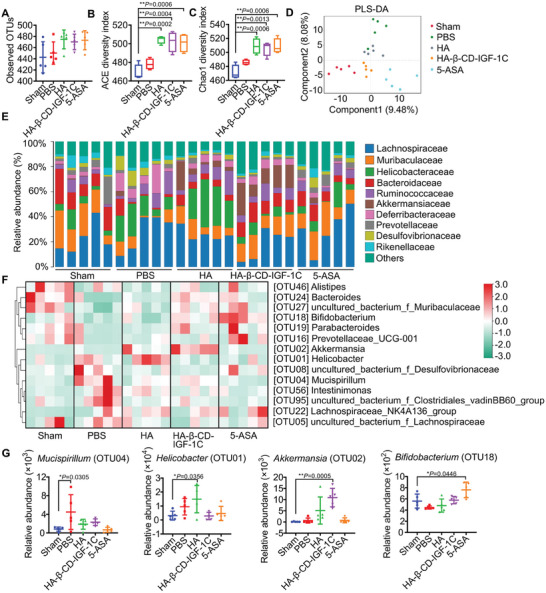
The supramolecular assembly of HA‐β‐CD‐IGF‐1C alters the composition of the gut microbiome. A) Assessment of the richness of observed bacterial operational taxonomic unit (OTU). B,C) Alpha diversity, including the ACE and Chao1 indices, in the mouse gut microbiota based on the OTUs of the indicated treatments. D) PLS‐DA analysis showing the beta diversity of the mouse gut microbiota based on the OTUs. Each point represents a sample, and each color indicates a treatment. E) Relative abundance of different bacteria at the family level. Bar plots showing the percentage of different families accounting for total sequences in the corresponding group. F) Heatmap of the relative abundance of different bacteria at OTU and genus levels. Cyan indicates less abundant, white represents intermediate abundance, and red represents the most abundant. G) Relative abundance of selected *Mucispirillum*, *Helicobacter*, *Akkermansia*, and *Bifidobacterium* at the genus level. All data are expressed as the mean ± s.d. The *P*‐values were calculated using one‐way ANOVA with Tukey's HSD multiple comparison post hoc test and defined as *P < 0.05 and **P < 0.01.

Recent evidence suggests that the gut microbiome functions as an ecosystem, providing pathogenic bacteria antagonism, immunoregulation, nutrient digestion, and metabolite production, and its dysfunction can lead to diseases directly or indirectly.^[^ ^16^ ^]^ To investigate whether supramolecular assembly of HA‐β‐CD‐IGF‐1C exhibits superior efficacy in modulating microbial diversity and composition, fecal 16S ribosomal RNA gene V3‐V4 region sequences were analyzed. We observed an increased operational taxonomic unit (OTU) richness in all groups, indicating that drug intervention significantly improved bacterial richness (**Figure** **5**A). Similarly, different alpha diversity indices, including ACE and Chao1, exhibited significantly greater tendencies in the HA, HA‐β‐CD‐IGF‐1C, and 5‐ASA groups (Figure 5B,C). We next examined beta diversity indices using partial least squares discriminant analysis (PLS‐DA), principal component analysis (PCA), principal coordinate analysis (PCoA), and nonmetric multidimensional scaling (NMDS). The results revealed a distinct gut microbiota pattern between HA‐β‐CD‐IGF‐1C and the other treatment groups (Figure 5D and Figure S12, Supporting Information). Next, we investigated the specific components that influence the profile of the gut microbiota at the family and class levels. All samples exhibited a similar composition of the gut microbiota but no similar relative abundance (Figure 5E and Figure S13A, Supporting Information). We performed a batch analysis of all groups at the genus level, and results revealed that the treatment with HA and HA‐β‐CD‐IGF‐1C enhanced the relative abundance of *Akkermansia* and *Bacteroides* (Figure S13B, Supporting Information). Through the combined analysis of the OTU results and relative abundances at the genus level, we found that mice with colitis exhibited increased relative abundances of the pro‐inflammatory bacteria *Mucispirillum* (OTU04) and *Desulfovibrio* (OTU08) (Figure 5F). We also observed that *Helicobacter* (OTU01), which is highly correlated with IBD, was enriched in PBS and HA‐treated mice but not in the Sham, HA‐β‐CD‐IGF‐1C‐ and 5‐ASA treated groups (Figure 5G). Moreover, HA‐β‐CD‐IGF‐1C treatment significantly increased *Akkermansia* abundance (OTU02) (Figure 5G), which is a next‐generation probiotic that protects mucus layer function, obesity, intestinal immunity, and tumorigenesis.^[^ ^17^ ^]^ In particular, the abundances of the probiotics *Prevotellaceae_UCG‐001* ^[^ ^18^ ^]^ (OTU16) and *Bifidobacterium* ^[^ ^19^ ^]^ (OTU18) and the common bacteria *Muribaculaceae* ^[^ ^20^ ^]^ (OTU27) were relatively higher in the 5‐ASA treatment group than in the HA and HA‐β‐CD‐IGF‐1C groups, suggesting that 5‐ASA could ameliorate colitis in mice in a particular pattern.

### 2.6 HA‐β‐CD‐IGF‐1C Alters the Structure and Function of the Bacterial Community

**Figure 6:**
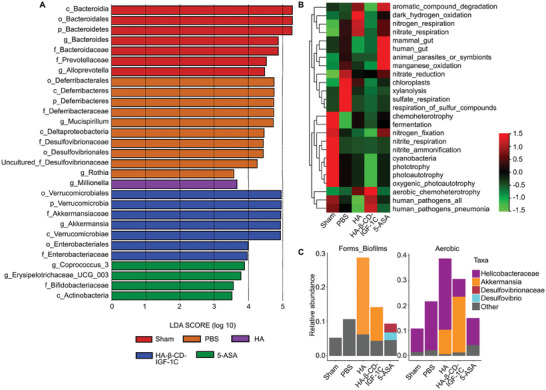
The supramolecular assembly of HA‐β‐CD‐IGF‐1C alters the predominant bacterial communities and functions of the gut microbiome. A) The representative bacterial community of the indicated treatment group was analyzed using the linear discriminant analysis (LDA) effect size (LEfSe) algorithm. The relative abundance of bacteria with a logarithmic LDA score > 3.5 was selected. B) The functional prediction of different bacterial communities in the corresponding group was calculated using the Functional Annotation of Prokaryotic Taxa (FAPROTAX) method. C) BugBase analysis illustrates the prediction of the microbial phenotype of different bacterial communities in the corresponding group.

To identify the specific biomarkers responsible for the modulation of the gut microbiota by HA‐β‐CD‐IGF‐1C, we performed a linear discriminant analysis (LDA) of effect size (LEfSe) to identify novel biological characteristics based on an LDA score >3.5. The genus *Bacteroides* (ranging from the class *Bacteroidia* to the family *Bacteroidaceae*) and the genus *Alloprevotella* (the family *Prevotellaceae*) were the typical gut microbial types of normal mice. Interestingly, the family *Deferribacteraceae* (ranging from the phylum *Deferribacteres* to the order *Deferribacterales*), the genus *Mucispirillum*, and the genus *uncultured_bacterium_f_Desulfovibrionaceae* (ranging from the order *Desulfovibrionales* to the family *Desulfovibrionaceae*) were identified as the dominant bacterial genera in colitis (**Figure** **6**A and Figure S14, Supporting Information). Furthermore, HA‐β‐CD‐IGF‐1C treatment favored the growth of the genera *Akkermansia* (ranging from the phylum *Verrucomicrobia* to the family *Akkermansiaceae*) and the family *Enterobacteriaceae* (the order *Enterobacteriales*), while 5‐ASA treatment promoted the genera *Coprococcus_3* and *Erysipelotrichaceae_UCG_003*, and the family *Bifidobacteriaceae*. These finds suggest that HA‐β‐CD‐IGF‐1C and 5‐ASA have different impacts on the composition of gut microbiota in mice with IBD.

We next used the Functional Annotation of Prokaryotic Taxa (FAPROTAX),^[^ ^21^ ^]^ Phylogenetic Investigation of Communities by Reconstruction of Unobserved States2 (PICRUSt2)^[^ ^22^ ^]^ and BugBase predictive algorithms to further assess the bacterial communities and potential functions. FAPROTAX analysis suggested that compared to sham and PBS treatments, the HA, HA‐β‐CD‐IGF‐1C and 5‐ASA treatments altered bacterial functions related to nitrogen respiration, nitrate respiration, aerobic chemoheterotrophy, human pathogens, mammalian gut and manganese oxidation (Figure 6B). In addition, the abundance of the genus *Akkermansia* (OTU02), which was highly enriched in the HA‐β‐CD‐IGF‐1C treatment group, was positively correlated with the terms “Forms_Biofilms” and “Aerobic” terms predicted by BugBase (Figure 6C). Interestingly, Clusters of Orthologous Groups of proteins (COG) analysis showed that the microbiota involved in our 16S rDNA sequencing results are closely related to cell wall/membrane/envelope biogenesis, energy production and conversion, amino acid transport and metabolism (Figure S15, Supporting Information). These finds suggest that HA‐β‐CD‐IGF‐1C can regulate the structure and functional homeostasis of the gut microbiota.

## 3 Discussion

**Figure 7:**
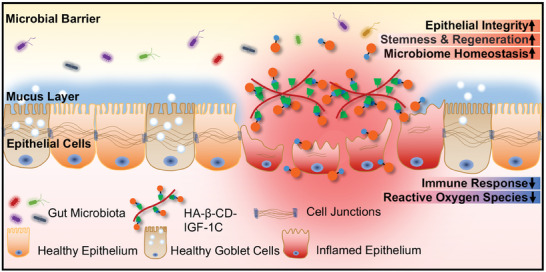
Schematic diagram depicting the therapeutic effects of HA‐β‐CD‐IGF‐1C in the IBD model. The HA‐β‐CD‐IGF‐1C enema serves as an artificial and biocompatible barrier that exhibits anti‐inflammatory and ROS inhibitory effects and further maintains the homeostasis of the intestinal flora via epithelial regeneration. Enema administration of the supramolecular assembly of HA‐β‐CD‐IGF‐1C simplified the drug delivery.

In the present study, we developed a rectal infusion supramolecular assembly of HA‐β‐CD‐IGF‐1C with good biocompatibility and anti‐inflammatory properties. Our results demonstrated that HA‐β‐CD‐IGF‐1C could ameliorate inflammatory infiltration, and further improve epithelial regeneration and intestinal barrier integrity. It also influenced the gut microbiota constitution with a significant increase in the abundance of the probiotic *Akkermansia*, suggesting enhanced intestinal microbiome homeostasis. These benefits can be attributed to the sustained release of IGF‐1C and maintains the integrity of the intestinal barrier (**Figure** **7**).

Currently available therapeutic strategies for IBD include 5‐ASA, glucocorticoids, monoclonal antibodies (McAbs) against tumor necrosis factor (TNF) and colectomy. However, most of these strategies aim to alleviate the secondary effects of IBD, and these approaches inevitably increase the risk of immune dysfunction, infection, renal toxicity, and hepatotoxicity rather than addressing the underlying causes related to intestinal barrier function and gut flora interactions.^[^ ^23^ ^]^ In this investigation, the enema administration of HA‐β‐CD‐IGF‐1C had greater effects on inflammation, ROS scavenging, epithelial regeneration, and gut microbiome regulation compared to 5‐ASA. Therefore, this supramolecular assembly could be a promising alternative for IBD patients after more detailed investigation on physiological and molecular mechanisms.

Our previous research demonstrated that the IGF‐1C peptide, which phosphorylates IGF‐1R, could be a potential substitute for IGF‐1.^[^ ^24^ ^]^ However, free IGF‐1C achieved limited antiapoptotic activity according to our results. This may be primarily caused by the short half‐life because of the lack of steric hindrance effect of the oligopeptide, despite it having functions similar to those of its full‐length form. In the present study, HA not only endows the supramolecular assembly with anti‐inflammatory functions but also prolongs IGF‐1C release. Furthermore, our study revealed markedly increased expressions of CD44 and LGR5. It is plausible that HA‐β‐CD‐IGF‐1C anchors more CD44^+^ cells via the HA‐CD44 interaction, subsequently promoting the survival and proliferation of LGR5^+^ intestinal stem cells via crypt‐targeted IGF‐1C delivery, which is consistent with previous report.^[^ ^25^ ^]^

High levels of ROS in the early stages of IBD contribute to the release of inflammatory cytokines, potentially leading to more severe forms of the disease. Long‐term infiltration of pro‐inflammatory cytokines and immune cells could result in a goblet cell deficiency and dysfunction of the bowel luminal epithelium. Therefore, removing these inflammatory molecules early on may be a key strategy to prevent further damage and complications. Here, we identified the supramolecular assembly of HA‐β‐CD‐IGF‐1C as a potential therapeutic agent for IBD that can reduce intestinal ROS levels. These results are consistent with previous conclusions that indicate that HA or IGF‐1C ameliorates ROS accumulation.^[^ ^26^ ^]^ Therefore, bioactive antioxidative HA incorporating an IGF‐1 mimicking peptide platform may be further applied to other oxidative and inflammatory diseases.

The intestinal epithelium comprises various types of tightly connected intestinal cells based on intercellular junction proteins and plays a vital role in maintaining internal immunoregulation separated from the external microbial environment. The pathogenesis of IBD is related to aberrant epithelial alterations, including decreased gap junctions and self‐renewal capacity. Our results demonstrated that HA‐β‐CD‐IGF‐1C significantly increased the expression of genes related to the gap junction and the integrity of the intestinal epithelium. Furthermore, our observations indicated that HA treatment also slightly increased junction protein expression, which is consistent with previous reports demonstrating that exogenous HA administration can strengthen barrier integrity and innate host defense by interacting with intestinal epithelium.^[^ ^26^, ^27^ ^]^

Recent evidence has shown that altered gut microbiota structure, which affects barrier function and adaptive immune responses, can contribute to the development of IBD. On the contrary, exceptional host‐microbe communications reflect the susceptibility and prognosis of IBD. In the present study, we performed 16S rDNA sequencing to determine whether enema administration of HA‐β‐CD‐IGF‐1C changes the intestinal microbiota composition and function in colitis mice. Consistent with previous studies,^[^ ^28^ ^]^ our analysis revealed that *Desulfovibrionaceae, Mucispirillum, Intestinimonas, Clostridiales*, and *Lachnospiraceae* were significantly enriched in the TNBS‐induced murine colitis model. The microbiological composition of the HA‐β‐CD‐IGF‐1C group showed a unique profile with enrichment of the *Akkermansia*, which is known to be related to intestinal epithelium integrity and ROS scavenging.^[^ ^29^ ^]^

Growing evidence suggests that HA‐based therapeutics hold promise for treating IBD by protecting the biophysical barrier and regulating intestinal permeability.^[^ ^26^, ^30^ ^]^ Given the convenience and good acceptance of enema administration, future investigations could expand to large animal models such as pigs and primates. Furthermore, HA‐β‐CD‐IGF‐1C has potential benefits for conjugating various anti‐inflammatory agents, such as curcumin and corticosteroids to the HA system through host–guest interactions. This feature significantly broadens the therapeutic scope of supramolecular designs, presenting the exciting prospect of developing effective combination therapies that not only target localized gastrointestinal inflammation, but also potentially extend to the treatment of systemic inflammatory conditions across multiple organ systems.

In conclusion, we developed and characterized an IGF‐1C delivery system utilizing an HA‐mediated guest–host interaction strategy for the treatment of IBD via rectal infusion. HA‐β‐CD serves to provide biocompatible steric hindrance and supramolecular interactions, facilitating the effective release of IGF‐1C. Our results demonstrate that the supramolecular assembly of HA‐β‐CD‐IGF‐1C exhibits superior efficacy compared to that of commercial drugs HA and clinically approved 5‐ASA, effectively restoring the integrity of intestinal barrier and modulating microbial composition and function. Collectively, the findings of this study provide a practical IBD therapeutic platform for multiple drug delivery and represent a notable step forward in the realm of preclinical translation of IGF‐1C based drugs.

## 4 Experimental Section

### Characterization of the Supramolecular Assembly of HA‐β‐CD‐IGF‐1C

The detailed preparation of the supramolecular assembly of HA‐β‐CD‐IGF‐1C can be found in the Supporting Information. The rheological properties were tested on an AR 2000ex (TA Instruments) system equipped with 25 mm parallel plates. For the frequency sweep test, 1 mL of HA, HA‐β‐CD, or HA‐β‐CD‐IGF‐1C at a concentration of 0.2 wt% was added at 37 °C to characterize the viscoelastic mechanical properties of HA‐β‐CD‐IGF‐1C. ITC was conducted in a PBS solution using a MicroCal PEAQ‐ITC titration microcalorimeter (Malvern). The host molecule HA‐β‐CD was used at a concentration of 20 × 10^−6^ m to titrate the guest molecule Ad‐IGF‐1C at a concentration of 200 × 10^−6^ m. Subsequently, data processing and analysis were performed using MicroCal PEAQ‐ITC Analysis Software (v1.22).

### Biocompatibility of the Supramolecular Assembly of HA‐β‐CD‐IGF‐1C

To optimize the concentrations of HA‐β‐CD‐IGF‐1C, the cell counting kit‐8 (CCK‐8) (Beyotime Biotechnology, Shanghai, China) was used. In brief, 5 × 10^3^ MODE‐K epithelial cells were seeded in 96‐well plates per well supplemented with different concentrations of HA‐β‐CD (0, 10, 20, 40, 80, 100, or 200 µg mL^−1^) or Ad‐IGF‐1C (0, 1, 2, 4, 8, 10, 50, 100, 200, or 400 µg mL^−1^) for 24 h. CCK‐8 reagent was added to each well and the plates were incubated for an additional 2 h at 37 °C. The optical density at 450 nm was detected to quantify cell proliferation.

### In Vivo Retention of the Supramolecular Assembly of HA‐β‐CD‐IGF‐1C

The colorectal distribution and retention of Ad‐Cy5.5 incorporated into HA‐β‐CD (HA‐(β‐CD‐Ad)‐Cy5.5) or dispersed in β‐CD (CD/Cy5.5) at a concentration of 200 µg mL^−1^ following intrarectal administration were investigated in BALB/c mice (8–10 weeks old). Briefly, mice were fasted for 24 h with free access to drinking water before subjected to rectal flushing with 200 µL of glycerol using a soft catheter 30 min before enema administration. Mice were sacrificed at 0.25, 1, 2, 6, and 12 h. The colorectal tracts were then harvested to evaluate fluorescence retention using an IVIS Lumina imaging system. In this study, BALB/c mice (8–10 weeks old) were purchased from the Laboratory Animal Center of the Academy of Military Medical Sciences (Beijing, China). All animal treatments and experimental procedures of the present study were approved by the Animal Experiments Ethical Committee of Nankai University and were in accordance with the National Institutes of Health (NIH) Guide for the Care and Use of Laboratory Animals (approval no. 20200022).

### TNBS‐Induced Colitis Model and HA‐β‐CD‐IGF‐1C Treatment

For the TNBS‐induced colitis model, the mice were fasted for 12 hours prior to modeling and then anesthetized with isoflurane (2%). Then 2.5% TNBS (w/v, Sigma Aldrich, USA) in 50% ethanol was slowly administered intrarectally to the rectum via a soft catheter. After an enema of the TNBS solution, the mice were kept in the vertical position for 3 min. Mice were anesthetized with isoflurane (2%), and then 200 µg mL^−1^ HA‐β‐CD conjugated with 10 µg mL^−1^ IGF‐1C was slowly administered into the colon lumen via a soft catheter after 24, 48, and 72 h of TNBS administration.

### BLI Imaging

To assess the ROS level in the colitis model, 0.01 m luminol (3‐aminophthalhydrazide, Sigma) dissolved in 0.1 m NaOH solution was administered via intraperitoneal injection. At the last time point of the whole‐body ROS imaging, the mice were sacrificed and the whole intestine was harvested. The bioluminescence signal was measured by an IVIS Lumina imaging system. The intensity of the signals was analyzed by Living Image software (Xenogen Corporation). The average radiance of the region of interest (ROI) in the abdominal region was quantified at different time points.

### Histology and Immunostaining

For histology, all mice were sacrificed and colorectal tissues were harvested at the indicated time points. Subsequently, the distal colorectums were harvested and gently cleaned with cold PBS to eliminate the fecal contents. All samples were placed in 4% paraformaldehyde (PFA) for 18 h, dehydrated with gradient ethanol, hyalinized with xylene, embedded in paraffin, and cut into 5 µm paraffin sections. For immunofluorescence staining, samples were fixed with 4% PFA for 18 h, dehydrated with a 30% sucrose solution overnight before being embedded in optimal cutting temperature (OCT) compound and cut into 5‐µm‐thick sections. Hematoxylin and eosin (H&E) staining and PAS staining were performed according to the standard protocol. Pathological score was assessed according to the presence of goblet cells, the depth of the intestinal crypt, the infiltration of inflammatory cells, and the degree of ulceration. For immunohistochemical and immunofluorescence staining, sections were incubated with primary antibodies against ZO‐1 (1:250; sc‐33725, Santa Cruz Biotechnology), OCLN (1:250; sc‐133256, Santa Cruz Biotechnology), E‐cad (1:250; #3195, Cell Signaling Technology), Ki67 (1:250; #9129, Cell Signaling Technology), and LGR5 (1:200; ab219107, Abcam) at 4 °C overnight, and the sections were incubated with HRP‐conjugated secondary antibodies or fluorescently labeled secondary antibodies at room temperature for 2 h. DAPI was used to visualize the nucleus.

### Microbiome 16S rDNA Sequencing and Bioinformatics Analysis

The intestinal fecal contents of the intestinal tract were collected, flash‐frozen in liquid nitrogen, and subsequently stored at −80 °C until the extraction of genomic DNA from the bacterial samples. Then, fecal genomic DNA was extracted from 0.25–0.5 g samples using a Magnetic Soil and Stool DNA Kit (TIANGEN, DP712) according to the manufacturer's instructions. 16S ribosomal DNA (16S rDNA) libraries covering V3‐V4 hypervariable regions were prepared using the primers 338F and 806R. The PCR products were purified using e.Z.N.A. TM Cycle‐Pure Kit (Omega) and qualified by Qsep‐400 before paired‐end sequencing on the Illumina NovaSeq 6000 platform according to the standard workflow of Biomarker Technology Co., Ltd. (Beijing, China).

For data analysis, the raw data were subjected to quality filtering using Trimmomatic (v0.33). Cutadapt (v1.9.1) was used to remove adapter sequences. Usearch (v10) was used to align and cluster OTUs at 97.0% similarity. UCHIME (v4.2) was used to detect and remove chimeras. Alpha diversity was used to evaluate the richness and complexity of each sample using indices including Chao1, Ace, Shannon, Simpson, and coverage in Quantitative Insights into Microbial Ecology (QIIME2) indices. Beta diversity analysis, including PCA, PCoA, NMDS, PLS‐DA, and heatmap analyses, was performed and plotted using the QIIME2 and R packages. The microbial characteristics were analyzed using LEfSe (http://huttenhower.sph.harvard.edu/lefse/) and visualized using a cladogram. For LEFSe analysis, an LDA score (log10) = 3.5 was used to assess the significance of biomarkers. PICRUSt2, BugBase, and FAPROTAX analyses were used to predict the differential microbial functions and phenotypes.

### Statistical Analysis

All experiments were carried out independently at least three times for each condition. Data are presented as mean ± standard deviation (S.D.). For comparisons between two groups, two‐tailed unpaired Student's *t*‐tests were used, while one‐way or two‐way analysis of variance (ANOVA) with post hoc Tukey's test was used for comparisons among more than two groups. Statistical analyses were performed using GraphPad Prism software. A *P*‐value <0.05 was considered to indicate statistical significance.

## Conflict of Interest

The authors declare no conflict of interest.

## Author Contributions

E.F. and M.Q. contributed equally to this work. Z.L., Q.Z., X.C., and E.F. conceived and designed the research. E.F. carried out most of the experiments and analyzed the data. Q.Z. and M.Q. contributed to the design and synthesis of HA‐β‐CD and Ad‐IGF‐1C. N.H., Y.Y., Y.L., Z.C.H., and Z.H. provided technical support. E.F., Y.Y., and Z.L. wrote the manuscript, and all authors approved the final version of this manuscript.

## Supporting information

Supporting Information

## Data Availability

The data that support the findings of this study are available from the corresponding author upon reasonable request. The gut microbiome sequence data has been deposited to NCBI (Bioproject PRJNA967099), https://www.ncbi.nlm.nih.gov/bioproject/PRJNA967099.
