# Supplementary material for: Biomimetic Supramolecular Assembly with IGF‐1C Delivery Ameliorates Inflammatory Bowel Disease (IBD) by Restoring Intestinal Barrier Integrity
Source: Adv Sci (Weinh). 2024 Jul 23;11(36):2403075. doi: 10.1002/advs.202403075 (PMC11423171; doi:10.1002/advs.202403075)
Supplement: Supplementary file 1 — Supporting Information [file ADVS-11-2403075-s001.docx]

**Supplementary Information**

**Supplementary Tables**

| DAI score | Weight loss | Stool consistency | Bleeding |
| --- | --- | --- | --- |
| 0 | < 1% | well-formed | OBT negative |
| 1 | 1-5% | semiformed and soft | OBT negative |
| 2 | 6-10% | liquid stools | OBT positive |
| 3 | 11-18% | diarrhea, slight bleeding | visible bleeding |
| 4 | > 18% | diarrhea, severe bleeding | gross bleeding |

**Table S1.** **Standards for the Evaluation of the Disease Activity Index (DAI).**

*OBT: fecal occult test

**Table S2.** **Primers used for RT-qPCR in this work.**

| **Gene** | **Forward primer sequence, 5’-3’** | **Reverse primer sequence, 5’-3’** |
| --- | --- | --- |
| *LGR5* | CCTACTCGAAGACTTACCCAGT | GCATTGGGGTGAATGATAGCA |
| *CD44* | CACCATTGCCTCAACTGTGC | TTGTGGGCTCCTGAGTCTGA |
| *IL-1α* | CGAAGACTACAGTTCTGCCATT | GACGTTTCAGAGGTTCTCAGAG |
| *IL-1β* | GCAACTGTTCCTGAACTCAACT | ATCTTTTGGGGTCCGTCAACT |
| *IL-4* | GGTCTCAACCCCCAGCTAGT | GCCGATGATCTCTCTCAAGTGAT |
| *IL-6* | TAGTCCTTCCTACCCCAATTTCC | TTGGTCCTTAGCCACTCCTTC |
| *IL-12* | CTGTGCCTTGGTAGCATCTATG | GCAGAGTCTCGCCATTATGATTC |
| *Caspase 3* | TGGTGATGAAGGGGTCATTTATG | TTCGGCTTTCCAGTCAGACTC |
| *TNF-α* | ACGGCATGGATCTCAAAGAC | AGATAGCAAATCGGCTGACG |
| *Gss* | CAAAGCAGGCCATAGACAGGG | AAAAGCGTGAATGGGGCATAC |
| *Gclc* | GGGGTGACGAGGTGGAGTA | GTTGGGGTTTGTCCTCTCCC |
| *ZO-1* | GCCGCTAAGAGCACAGCAA | TCCCCACTCTGAAAATGAGGA |
| *OCLN* | TTGAAAGTCCACCTCCTTACAGA | CCGGATAAAAAGAGTACGCTGG |

**Supplementary Methods**

**Preparation of HA-β-CD-IGF-1C**

HA-β-CD was synthesized following a previously established protocol[1]. Briefly, for the preparation of HA-TBA, 2 g of HA (mw = 80 kDa) was completely dissolved in 100 ml of deionized water and 6 g of Dowex 50 W×8 was added to the HA solution and stirred for 30 min at RT. The HA solution was then neutralized to pH 7.02–7.05 by adding tetrabutylammonium hydroxide (TBA-OH) and then lyophilized.

For the preparation of HA-β-CD, 10 g of β-cyclodextrin was suspended in 70 ml of deionized water and cooled to 0 °C. A total of 2.1 g of p-toluenesulfonyl chloride was dissolved in 5 ml of acetonitrile and added dropwise to the solution. The reaction mixture was stirred at room temperature for 2 h. Then 8 g of ammonium chloride was added to the solution to adjust the pH to 9, the solution was cooled on ice, and the precipitate was collected under vacuum pressure overnight to obtain a dry CD-Tos product. The flask was charged with 1 g of CD-Tos, 4 g of 1,6-hexanediamine (HDA) and 5 ml of N, N-dimethylformamide (DMF). The reaction was carried out under nitrogen at 80 °C for 18 h. The product was precipitated from cold acetone, washed with cold diethyl ether, and dried under vacuum to afford CD-HDA. HA-TBA (1 g) and CD-HDA (1.2 g) were added to the ﬂask and purged with nitrogen before adding 40 ml of anhydrous DMSO. The mixture was stirred until it was fully dissolved, and 0.4 g of BOP was dissolved in 10 ml of anhydrous DMSO added to a solution of HA-TBA/CD-HDA and stirred for 3 h at RT. The mixture was transferred to dialysis tubes and dialyzed for 5 days at RT, after which the water was changed twice daily. The solution was frozen and lyophilized to provide the final product HA-β-CD. The chemical structure of HA-β-CD was characterized by NMR (^1^H NMR, Bruker, AVANCE III) using D_2_O as the solvent.

For the preparation of adamantane-modified IGF-1C (Ad-IGF-1C), peptides were synthesized according to the standard Fmoc-solid-phase peptide synthesis method and purified by HPLC. Briefly, the C-terminus of threonine (T) was attached to the resin at a loading rate of 0.5 to 0.7 mmol/g, and the amino Fmoc group was removed using 25% piperidine (C_5_H_11_N) dissolved in DMF. Then activated glutamine (Q), proline (P), alanine (A), arginine (R), serine (S), serine (S), serine (S), glycine (G), tyrosine (Y), glycine (G) and 1-amantanoacetic acid were repeatedly added for dehydration and condensation reactions under catalysis of hexafluorophosphate benzotriazole tetramethyl uranium (HBTU) and N,N-diisopropylethylamine (DIEA). The resin was successively rinsed 10 times with 30 ml of DMF and treated with trifluoroacetic acid (TFA) to elute the peptides from the resin. Then Ad-IGF-1C was purified by HPLC.

To generate supramolecular assemblies of HA-β-CD-IGF-1C, HA-β-CD and Ad-IGF-1C dissolved in cold PBS were mixed for 2 h in ice water on a magnetic stirrer. The resulting HA-β-CD-IGF-1C was diluted in PBS or culture medium for *in vitro* and *in vivo* experiments.

**Measurement of the remaining and released IGF-1C**

For the detection of IGF-1C release *in vitro*, 200 μl of supramolecular assemblies of HA-β-CD-IGF-1C or the mixture of HA and IGF-1C were deposited at the bottom of a 1.5 ml protease-free Eppendorf tube and covered with a volume of 200 μl of 1×PBS solution. Then 100 μl of the supernatant solution was collected at different time points and replaced with 100 μl of fresh 1×PBS. Free IGF-1C treated with DMSO or HA treated with CPY was used as a positive control or a negative control, respectively. The concentration of IGF-1C was detected using a Micro BCA protein assay kit (23250, Thermo Scientific) according to the manufacturer’s instructions.

To measure the amount of IGF-1C that remains after CPY treatment *in vitro*, 100 μl of HA-β-CD-IGF-1C or a mixture of HA and IGF-1C was added to the bottom of a 1.5 ml protease-free Eppendorf tube supplemented with 1 mg/ml CPY. The mixture was incubated at RT for up to 24 h, and the IGF-1C concentration was detected by a Micro BCA protein assay kit according to the manufacturer’s instructions.

**Cell culture**

The mouse intestinal epithelial MODE-K cell line was cultured in a humidified atmosphere under 5% CO_2_ at 37 °C in 1640 medium (HyClone) supplemented with 10% fetal bovine serum (FBS, Biological Industries, Israel), 1% penicillin–streptomycin solution (Gibco, Grand Island, NY), and 1% L-glutamine (Gibco). The human colon epithelial cancer cell line Caco-2 was cultured under the same air and temperature conditions in MEM (Biological Industries) supplemented with 15% FBS (Biological Industries), 1% penicillin–streptomycin solution (Gibco), 1% L-glutamine (Gibco), and 1% non-essential amino acids (Gibco). The HEK293T cell line was cultured in Dulbecco’s modified Eagle’s medium (DMEM, Gibco) under the same conditions as MODE-K cells.

**Dextran sulfate sodium (DSS)-induced mouse UC model**

For the DSS-induced ulcerative colitis (UC) model, 2.5% DSS (w/v) was dissolved in mouse drinking water ad libitum for 1 week. Mouse feces were tested using a urine fecal occult blood test kit to assess the severity of UC.

**Measurement of inflammatory molecule production in the colon**

The samples (100 mg) were cut from the colon tissue and washed and homogenized in cold PBS before centrifugation at 4 ℃ for 15 min. The supernatant was collected to measure the levels of the cytokines IL-4, IL-10, TNF-α, IL-1β, and IL-6 by using ELISA kits (PI613, PI513, PT513, PI301, PI326; Beyotime Biotechnology, Shanghai, China) following the manufacturer’s instructions.

**Generation of the double fusion (DF)-MODE-K cell line**

For the construction of the double fusion (DF) reporter lentiviral of firefly luciferase and green fluorescence protein (Fluc-GFP), HEK293T cells were transfected at 70% confluence with 12 μg of p-VSVG, psPAX-2 and DF reporter-expressing vectors with Lipofectamine 2000 (Thermo Scientific), and the supernatant containing the lentiviral particles was collected and condensed. MODE-K cells were then infected with DF lentivirus and selected using an inverted fluorescence microscope with Alexa Fluor 488 (Olympus IX53) to generate the stable DF-MODE-K cell line.

**Bioluminescence imaging (BLI) analysis of cells**

For cell BLI analysis, different numbers of intestinal cells were seeded in a 6-well plate to construct a standard curve as previously described. Briefly, 1 mg/ml D-luciferin was added to the cell culture medium and imaged for 1 min using an IVIS Lumina imaging system. The average radiance of the ROI was calculated by Living Image software. To investigate the bioprotective function of HA-β-CD-IGF-1C, 5×10^4^ DF-MODE-K cells were seeded into 24-well plates per well. H_2_O_2_ was added at different final concentrations (0, 0.3, 0.5, 1, 2, and 5 mM) and incubated for 6 hours, followed by BLI analysis.

**Dichlorodihydrofluorescein diacetate (DCFH-DA) staining and flow cytometry**

To assess the ROS scavenging effect of HA-β-CD-IGF-1C, intestinal MODE-K cells were pretreated with 500 μM H_2_O_2_ or PBS for 8 h. Then each well was washed 3 times with PBS and further incubated with HA, HA-β-CD-IGF-1C or 5-ASA for another 6 h. After treatment, 10 μM DCFH-DA (Beyotime Biotechnology, Shanghai, China) was added to 1640 medium and incubated at 37 °C for 1 h. Subsequently, all cells were trypsinized and resuspended in PBS. Fluorescence signals of fluorescent 2’,7’-dichlorofluorescein (DCF) generated from DCFH-DA by oxidation were monitored by flow cytometry in an AlexaFluor-488 channel of an FACSCalibur (BD Biosciences, San Jose, CA), and the data were analyzed by FlowJo software (Tree Star, San Carlos, CA).

**Real-time quantitative PCR (RT-qPCR)**

Total RNA was extracted from cells or colon tissue using TRIzol reagent (Invitrogen) according to the manufacturer’s instructions. A total of 2 μg cDNA was subjected to reverse transcription by the First-Strand cDNA Synthesis System (TransGen Biotech). Quantitative PCR was performed using Hieff qPCR SYBR Green Master Mix (Yeasen) on a CFX96 Touch System (Bio-Rad, Hercules, CA).

**Western blot**

The mouse colons were lysed in RIPA lysis buffer containing β-mercaptoethanol and a proteinase inhibitor cocktail on ice for 30 min according to the manufacturer’s protocol. Subsequently, a BCA protein assay kit was used to measure the protein concentration. Protein samples were loaded onto a 10% SDS-PAGE gel and electroblotted onto a polyvinylidene fluoride (PVDF) membrane. Subsequently, the membranes were blocked with 5% fat-free milk for 2 h and incubated with primary antibodies against β-tubulin (1:10,000; 10068-1-AP, Proteintech), LGR5 (1:1000; ab219107, Abcam), CD44 (1:1000; ab243894, Abcam), Caspase3 (1:1000; WL02117, Wanleibio), Cleaved Caspase3 (1:1000; WL01992, Wanleibio), ZO-1 (1:1000; sc-33725, Santa Cruz Biotechnology) and OCLN (1:1000; sc-133256, Santa Cruz Biotechnology). The HRP-linked secondary antibodies were incubated with the membranes and the HRP signal was detected using an HRP substrate.

***In vitro* intestinal permeability assay**

The transepithelial electrical resistance (TEER) of the Caco-2 monolayers was measured and 4 kDa fluorescein isothiocyanate (FITC)-dextran (FD4) was used according to the methods described previously [2]. In brief, 5×10^5^ Caco-2 cells were seeded in a 0.4 μm Transwell insert (polycarbonate membrane, 24-well, NEST). The TEER value was monitored weekly for 3 weeks to ensure monolayer confluence and epithelization (TEER > 500 Ω/cm^2^). After treatment with 1 mM H_2_O_2_ for 6 h, a total volume of 200 μl of FD4 (1 mg/ml) in MEM was added to the apical chambers of inserts containing PBS- or HA- or HA-β-CD-IGF-1C- or 5-ASA-pretreated Caco-2 monolayers. After incubation for 2 hours at 37 ℃ under 5% CO_2_, the basolateral medium was collected and measured at wavelengths of 490/520 nm (Ex/Em) on a Synergy H4 Multi-Mode Microplate Reader. The FD4 concentration was calculated on the basis of a standard curve constructed using blank MEM medium.

***In vivo* intestinal permeability assay**

For the *in vivo* intestinal permeability assay, all groups of mice fasted for 6 hours before FD4 (0.5 mg/kg) oral gavage. The mouse intestinal signal at 488 nm was collected and measured by an IVIS Lumina imaging system. The signal intensity was analyzed by Living Image software (Xenogen Corporation). Murine serum samples were collected from each group 4 h after FD4 oral gavage, and fluorescence was measured on a Synergy H4 Multi-Mode Microplate Reader. The concentration of FD4 was calculated based on a standard curve generated using blank mouse serum.

**Supplementary Figures and Legends**

**
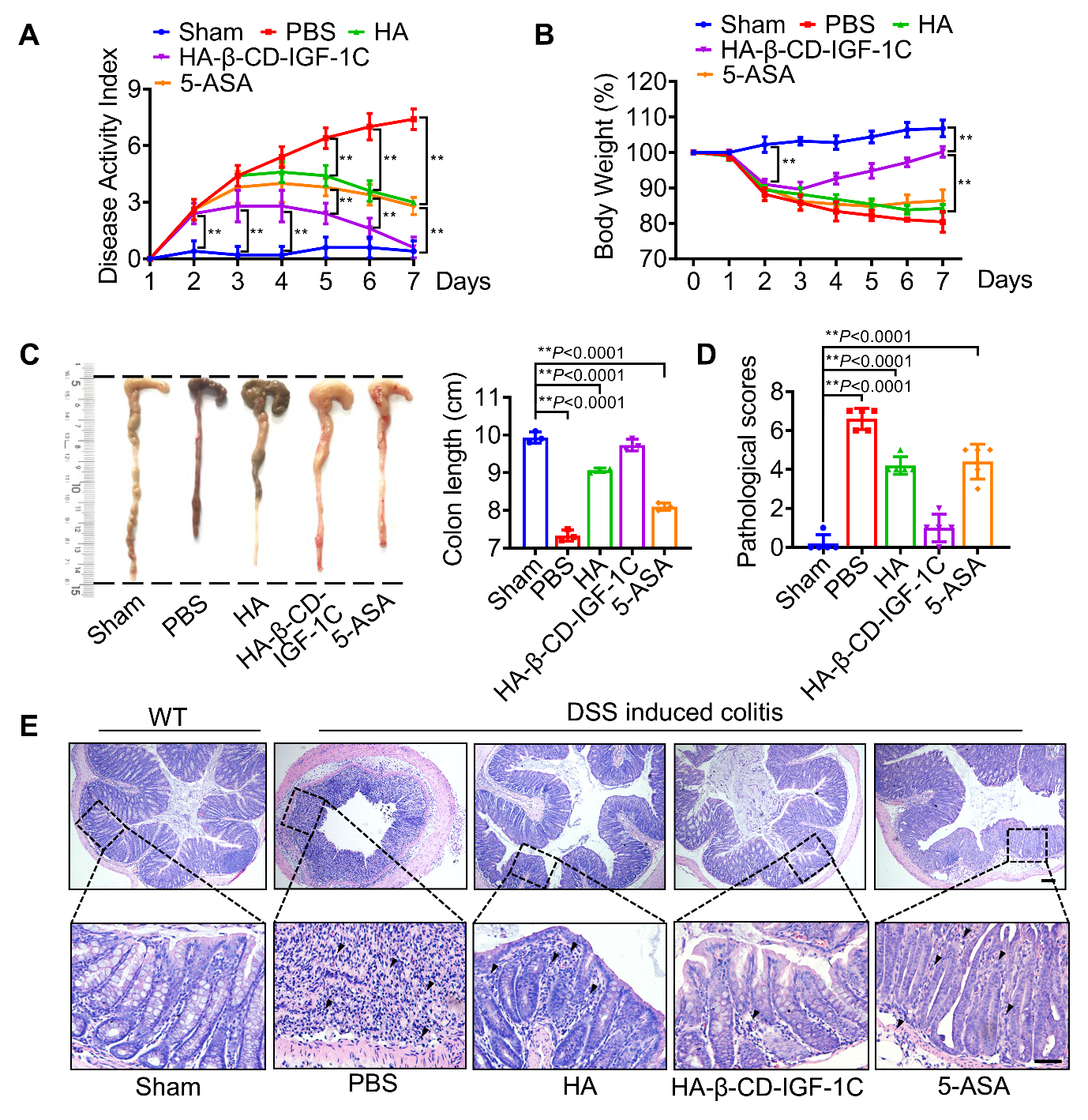
**

**Figure S1. Effect of** **HA-β-CD-IGF-1C on the DSS-induced UC model. (A, B)** The DAI score **(A)** and the percentage of change in body weight **(B)** of healthy or UC mice. **(C)** Representative images of the appearance and colon length. **(D, E)** Histological H&E images **(E)** of colon tissue from DSS-induced UC mice after different treatments. Pathological scores **(D)** were determined according to the degree of intestinal goblet cell/crypt damage and inflammatory infiltration in the mucosa, submucosa, and basal lamina. Black arrows: inflammatory sites. Scale bar: 100 μm. All data are expressed as mean ± s.d. The *P* values were calculated using one-way ANOVA with Tukey’s HSD multiple comparison post hoc test and defined as * *P* < 0.05, ** *P* < 0.01.


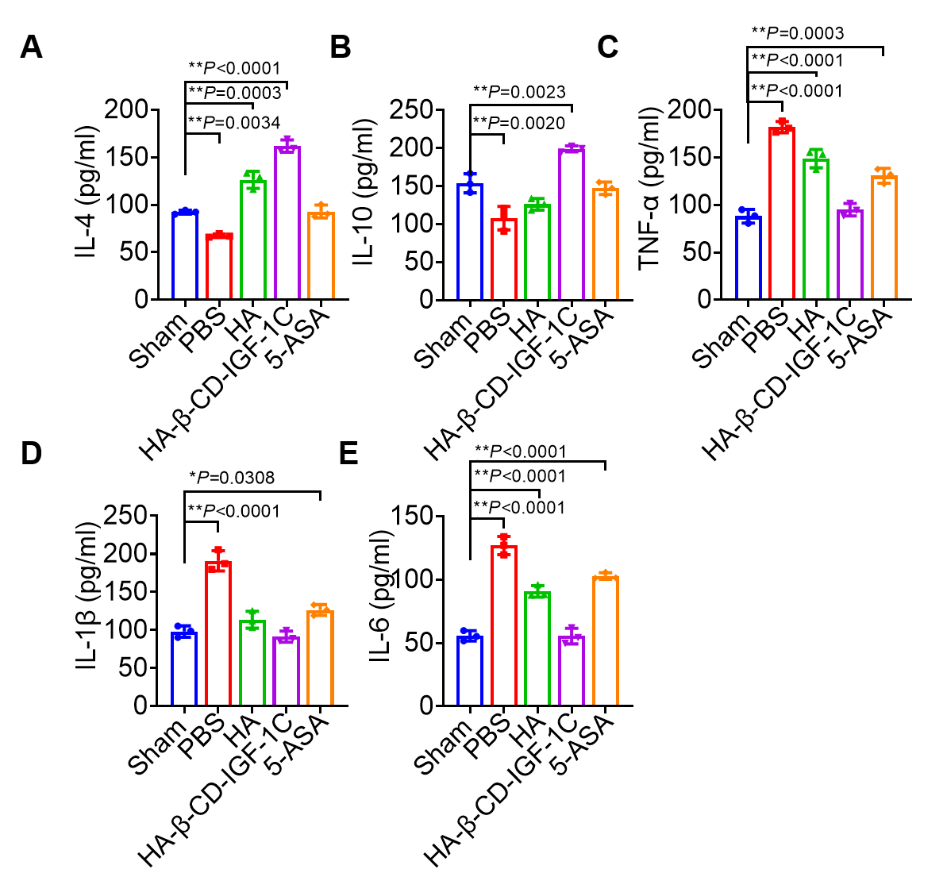


**Figure S2.** **The production of inflammatory cytokines, including IL-4, IL-10, TNF-α, IL-1β and IL-6, in the colons of the mice was measured by ELISA. (A)** IL-4. **(B)** IL-10. **(C)** TNF-α. **(D)** IL-1β. **(E)** IL-6. All data are expressed as mean ± s.d. The *P* values were calculated using one-way ANOVA with Tukey’s HSD multiple comparison post hoc test and defined as **P* < 0.05, ***P* < 0.01. Scale bar, 100 μm.


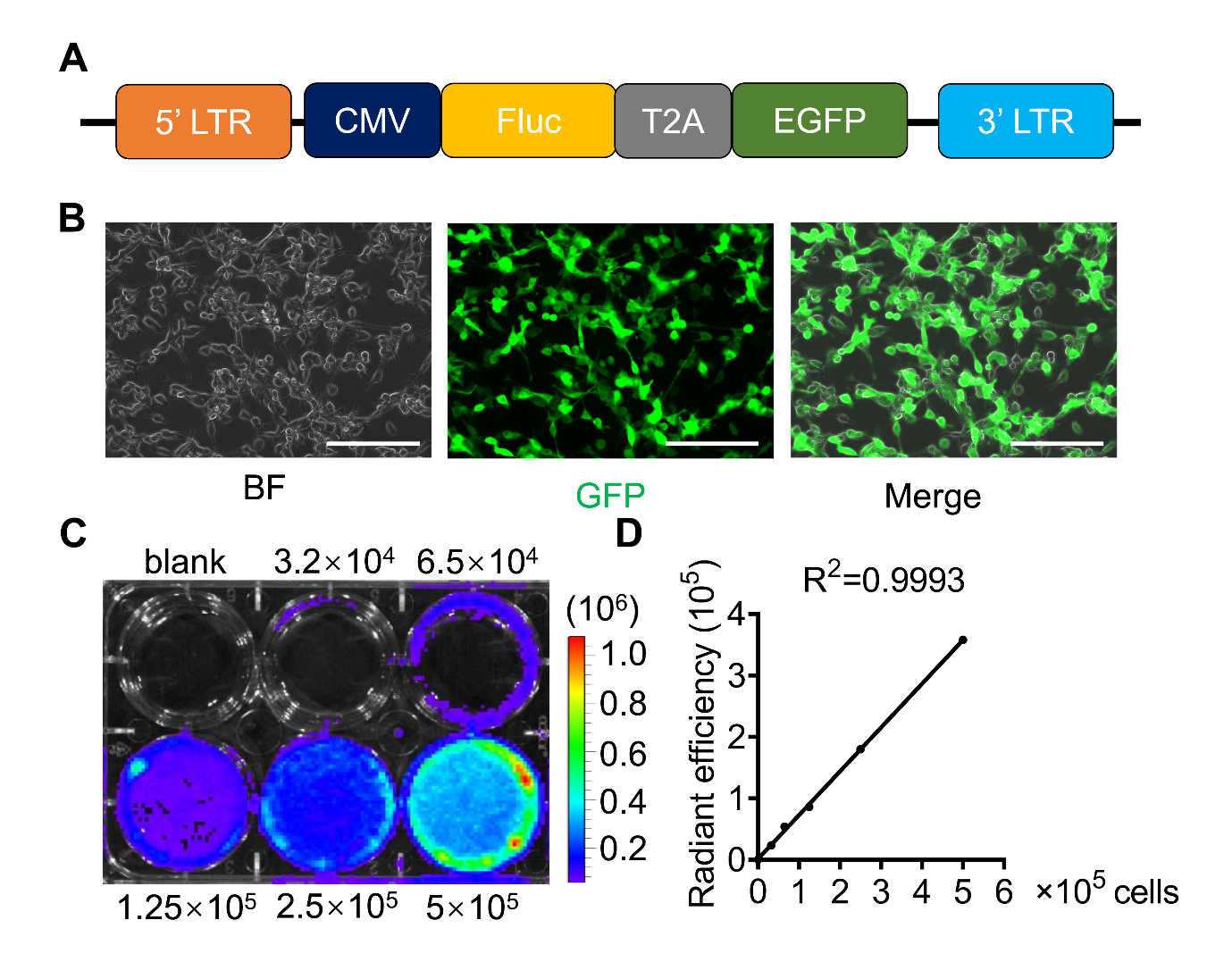


**Figure S3.** **Generation and characteristic of the DF-MODE-K intestinal cell line. (A)** Schematic representation of a firefly luciferase and a green fluorescence protein (Fluc-GFP) double fusion (DF) reporter gene in the MODE-K cell line. **(B)** MODE-K cells strongly expressed GFP. **(C, D)** BLI quantification demonstrated a significant linear relationship between the number of DF-MODE-K cells and the average radiance of Fluc.


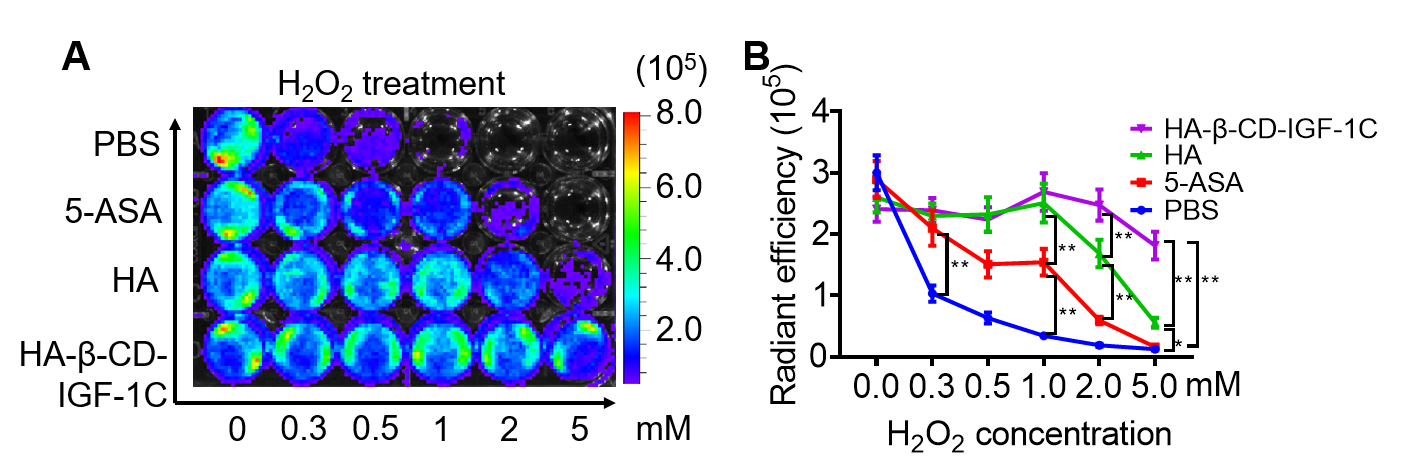


**Figure S4. Supramolecular assembly of** **HA-β-CD-IGF-1C ameliorates cell apoptosis of DF-MODE-K intestinal cells treated with H_2_O_2_.** **(A, B)** BLI **(A)** and quantitative analysis **(B)** were used to assess DF-MODE-K cell survival. Cells were cultured on noncoated, 5-ASA-coated, HA-coated and HA-β-CD-IGF-1C-coated plates under exposure to H_2_O_2_ for 6 h. All data are expressed as mean ± s.d. The *P* values were calculated using one-way ANOVA with Tukey’s HSD multiple comparison post hoc test and defined as **P* < 0.05, ***P* < 0.01. Scale bar, 100 μm.


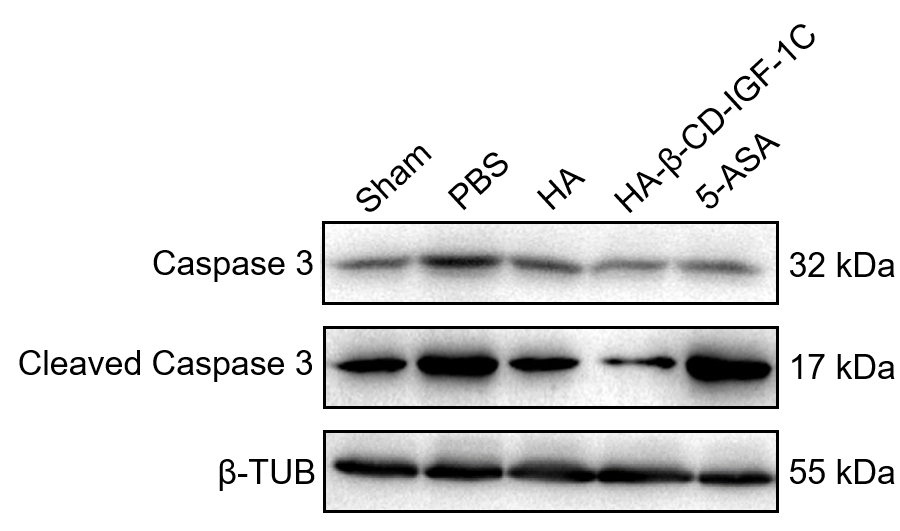


**Figure S5. Western blot analysis of Caspase3 and Cleaved Caspase3 in mouse epithelium under the indicated conditions.** β-TUBULIN was included as a loading control.


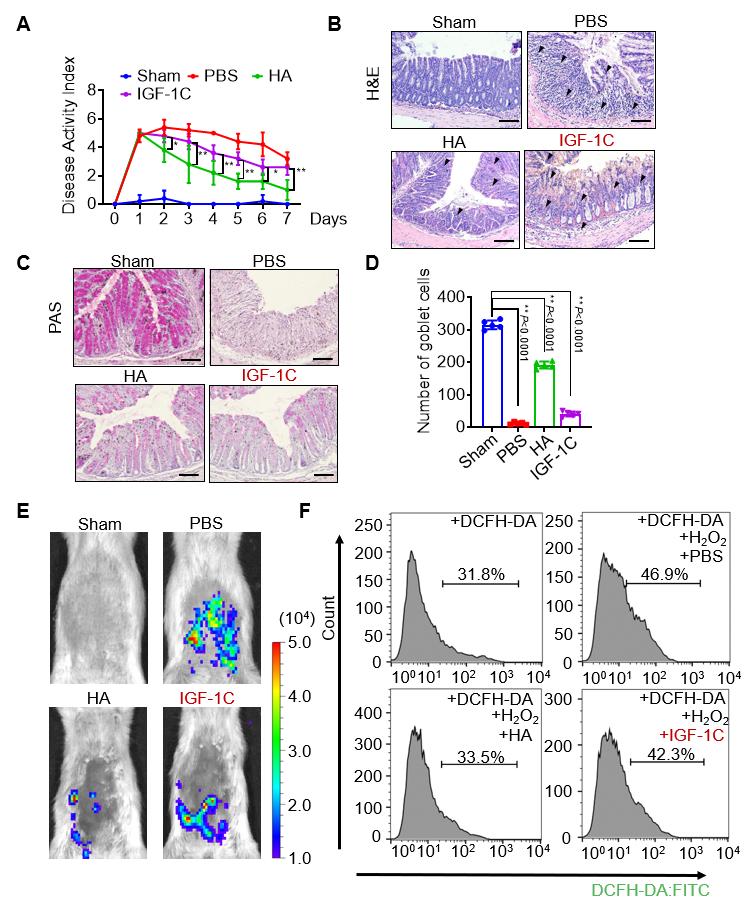


**Figure S6. Therapeutic efficacy of IGF-1C** **in both animal experiments and ROS scavenging assays. (A)** DAI scores of healthy or colitis mice subjected to different treatments. **(B)** Histological images of colon tissue from mice after different treatments. Arrows: inflammatory sites. **(C, D)** PAS staining images **(C)** and quantitative analysis **(D)** illustrating the number of goblet cells in the colonic tissue of mice after different treatments. **(E)** ROS activity in colitis mice was tracked by the BLI system on day 7 after different treatments. **(F)** The oxidized DCF fluorescence of MODE-K cells after treatment and H_2_O_2_ exposure was measured by flow cytometry. All data are expressed as mean ± s.d. The *P* values were calculated using one-way ANOVA with Tukey’s HSD multiple comparison post hoc test and defined as **P* < 0.05, ***P* < 0.01. Scale bar, 100 μm.


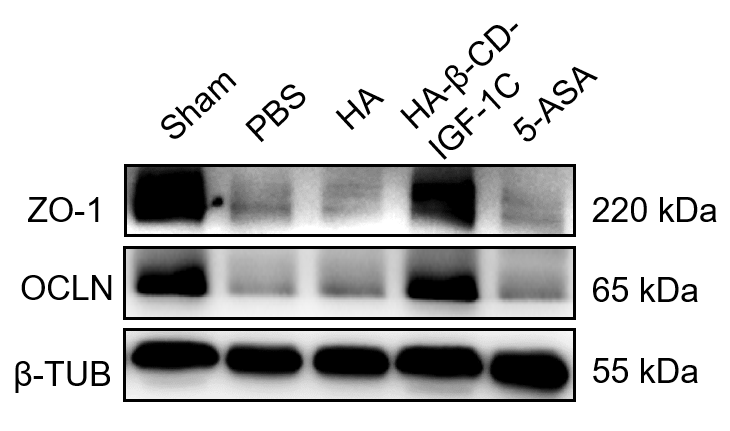


**Figure S7. Western blot analysis of ZO-1 and OCLN in the mouse epithelium under the indicated conditions.** β-TUBULIN was included as a loading control.


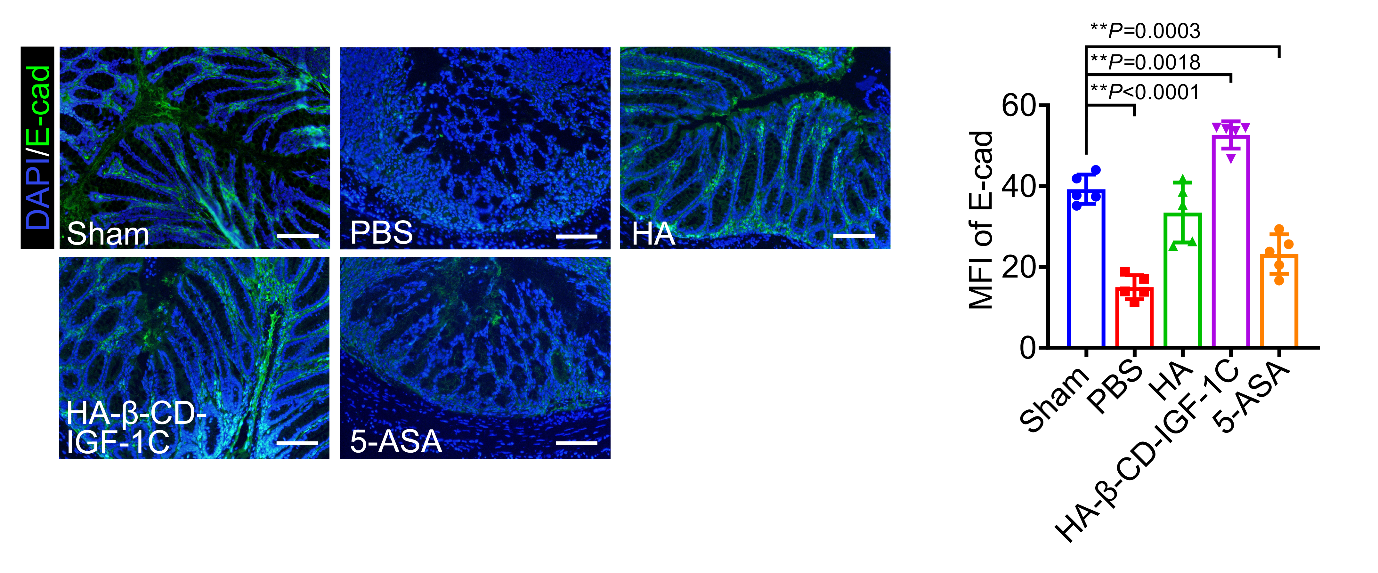


**Figure S8.** **Representative images of the epithelial marker E-cadherin show the gut re-established the function.** Cell nuclei were stained with DAPI (blue). All data are expressed as mean ± s.d. The *P* values were calculated using one-way ANOVA with Tukey’s HSD multiple comparison post hoc test and defined as **P* < 0.05, ***P* < 0.01. Scale bar, 100 μm.


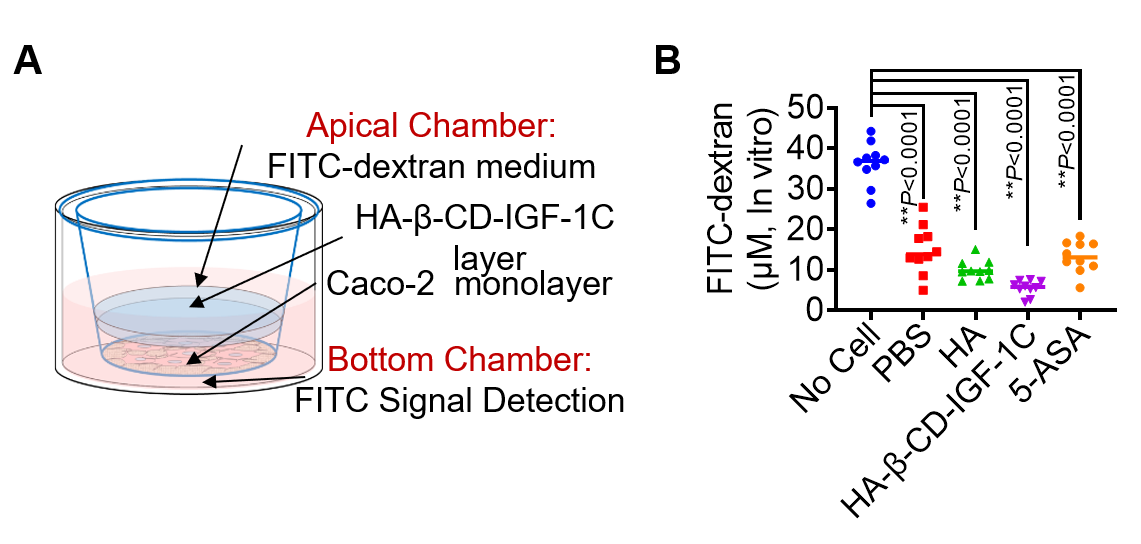


**Figure S9. The supramolecular assembly of HA-β-CD-IGF-1C contributes to the integrity of the intestinal epithelial *in vitro*. (A)** Experimental design to determine the effect of HA-β-CD-IGF-1C on epithelial permeability through the development of an *in vitro* model of differentiated Caco-2 monolayers. **(B)** FD4 filtered across the cell monolayers was used for signal measurement in the basolateral chamber after TEER value monitoring. All data are expressed as mean ± s.d. The *P* values were calculated using one-way ANOVA with Tukey’s HSD multiple comparison post hoc test and defined as **P* < 0.05, ***P* < 0.01. Scale bar, 100 μm.


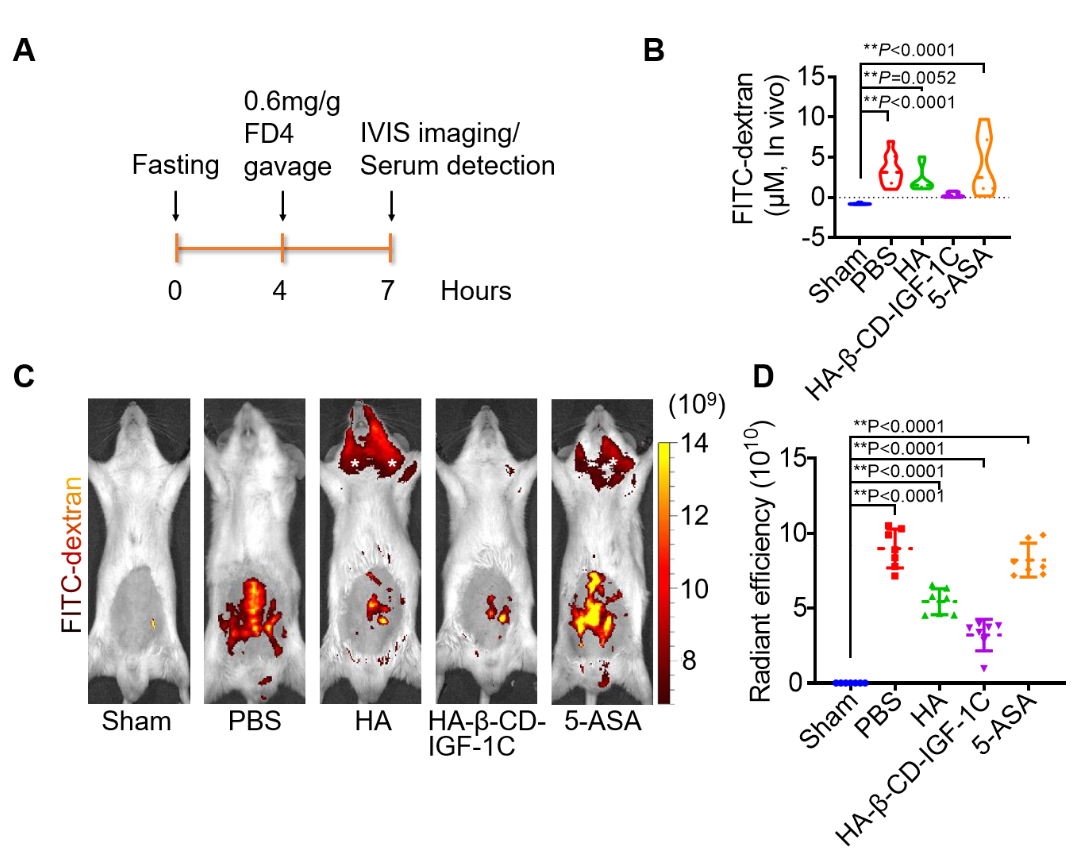


**Figure S10. The supramolecular assembly of HA-β-CD-IGF-1C contributes to the integrity of the intestinal epithelial *in vivo*. (A)** Experimental design to determine the effect of HA-β-CD-IGF-1C on epithelial permeability *in vivo***. (B)** The FD4 signal in the blood was tracked by a microplate reader after 3 days of the indicated treatments. **(C, D)** *In vivo* FD4 images **(C)** and quantitative analysis **(D)** of the abdominal region in colitis mice tracked by the IVIS Lumina imaging system *in vivo* after 3 days of indicated treatments. White asterisks: nonspecific signals located in the oral cavity and pharynx. All data are expressed as mean ± s.d. The *P* values were calculated using one-way ANOVA with Tukey’s HSD multiple comparison post hoc test and defined as * *P* < 0.05, ** *P* < 0.01. Scale bar, 100 μm.

**
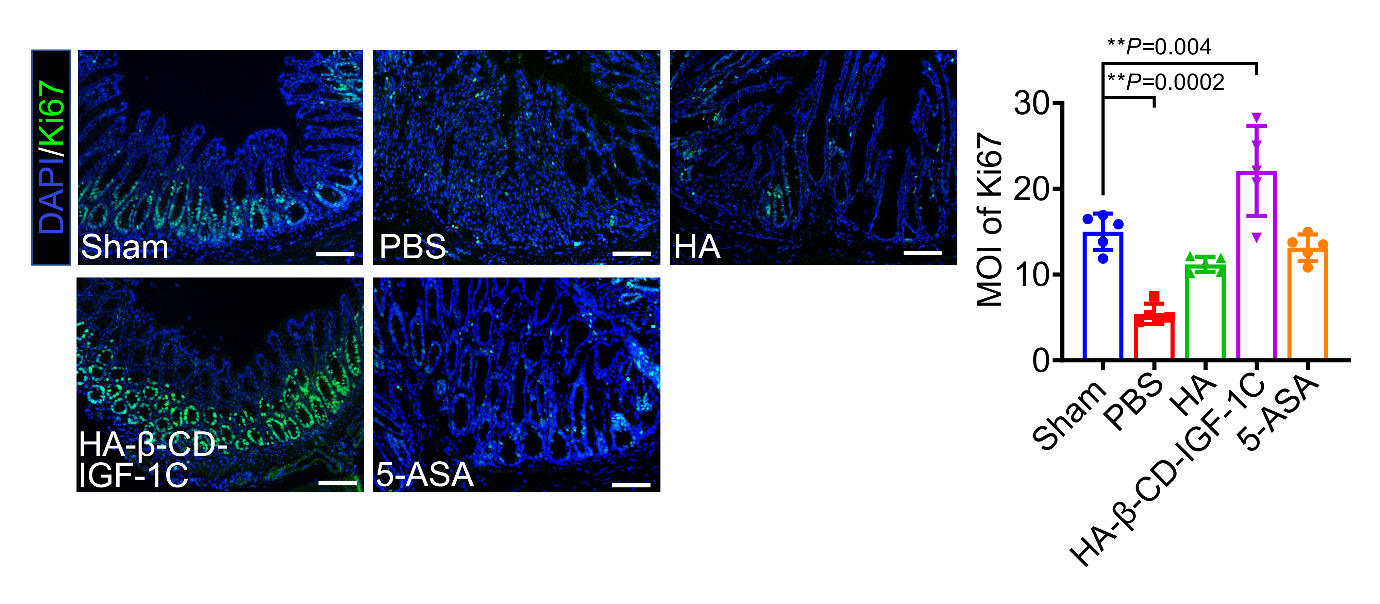
**

**Figure S11.** **Representative immunofluorescence images of Ki67 staining.** Cell nucleus were stained with DAPI (blue). Representative images of six slides from three independent experiments are shown. All data are expressed as mean ± s.d. The *P* values were calculated using one-way ANOVA with Tukey’s HSD multiple comparison post hoc test and defined as * *P* < 0.05, ** *P* < 0.01. Scale bar, 100 μm.


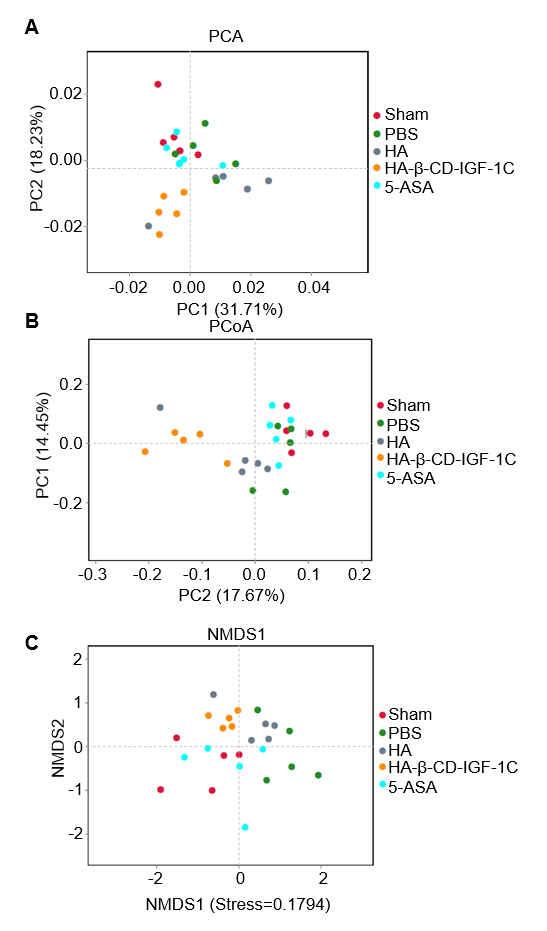


**Figure S12.** **PCA, PCoA, and NMDS analysis showing the beta diversity of the mouse gut microbiota based on OTUs.** Each point represents a sample, and each color indicates a treatment.


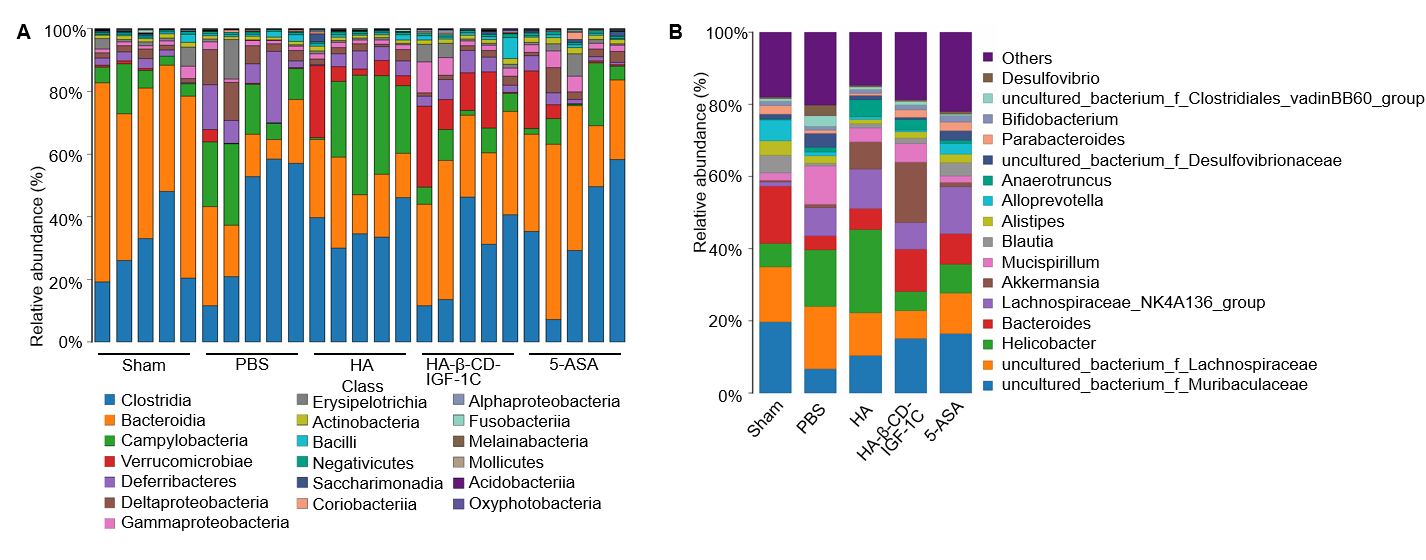


**Figure S13. The supramolecular assembly of HA-β-CD-IGF-1C significantly alters the diversity and composition of the gut microbiota. (A)** Relative abundance of different bacteria at the class level. Bar plots showing the percentage of different classes and genera that represent the total sequences in the corresponding group. **(B)** Relative abundance of different bacteria at the genus level. Bar plots showing the percentage of different classes and genera accounting for the total sequences in the corresponding group.


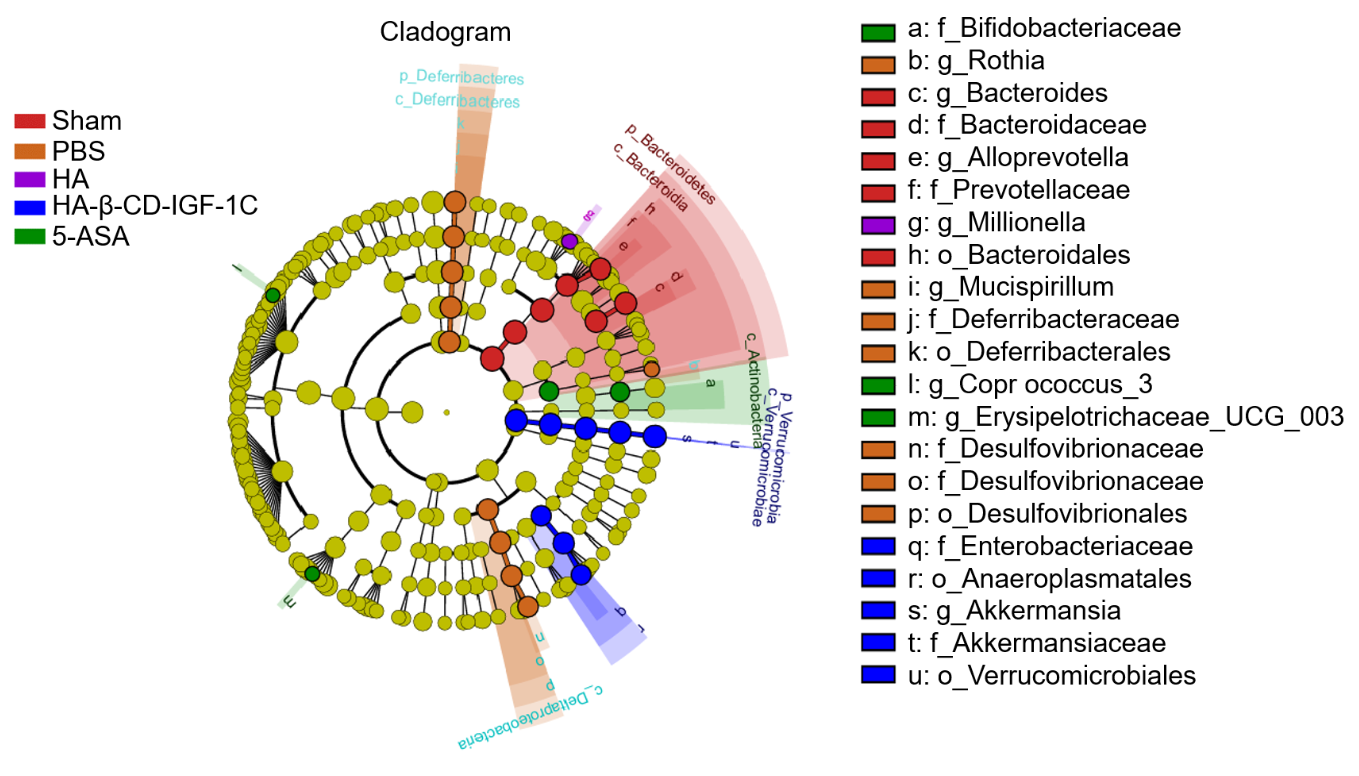


**Figure S14.** **The supramolecular assembly of HA-β-CD-IGF-1C changes the structure of the microbial community by altering the abundance of *Akkermansia*.** Cladogram diagram of LEfSe analysis depicting the taxonomic association from phylum to species in the corresponding group. Each circle represents a specific taxonomic rank. Taxonomic features that were not significantly different are colored yellow, and different colors indicate different treatments.


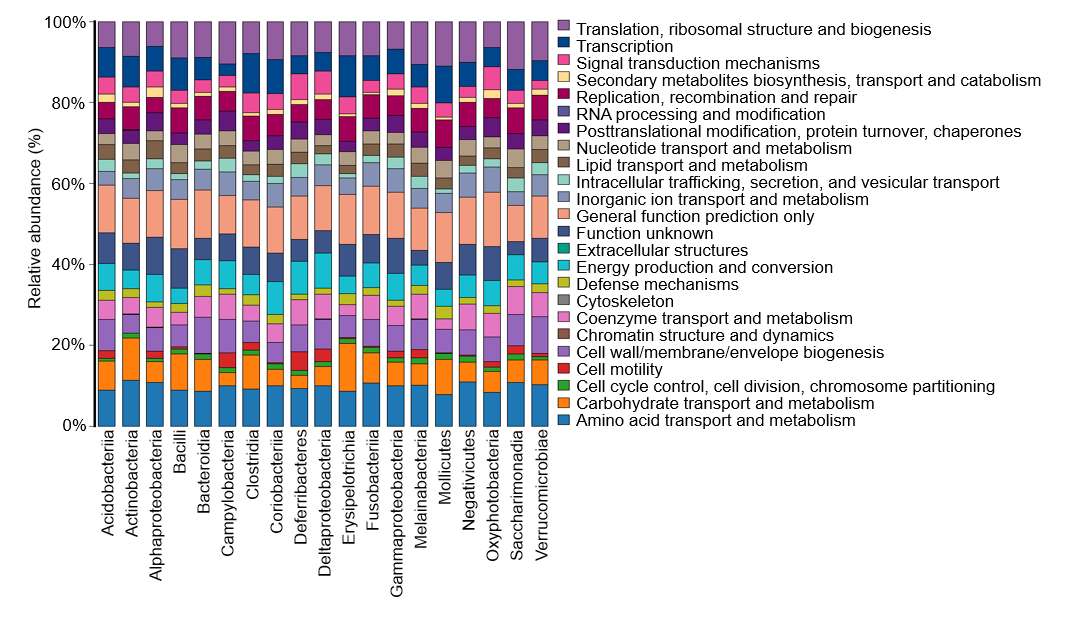


**Figure S15. PICRUSt2 bar plots indicating the bacterial communities and potential functions according to the Clusters of Orthologous Groups (COG) annotations.**

**References**

[1] C. Loebel, C. B. Rodell, M. H. Chen, J. A. Burdick, *Nat Protoc* **2017**, *12* (8), 1521, <https://doi.org/10.1038/nprot.2017.053>.

[2] a) S. He, Y. Guo, J. Zhao, X. Xu, N. Wang, Q. Liu, *Front Pharmacol* **2020**, *11*, 376, <https://doi.org/10.3389/fphar.2020.00376>; b) R. Yang, Q. Hui, Q. Jiang, S. Liu, H. Zhang, J. Wu, F. Lin, K. O, C. Yang, *Antioxidants (Basel)* **2019**, *8* (8), <https://doi.org/10.3390/antiox8080250>.
